# Variability and distribution of the golden-headed weevil *Compsusauricephalus* (Say) (Curculionidae: Entiminae: Eustylini)

**DOI:** 10.3897/BDJ.8.e55474

**Published:** 2020-07-09

**Authors:** Jennifer C. Girón, M. Lourdes Chamorro

**Affiliations:** 1 Natural Science Research Laboratory, Museum of Texas Tech University, Lubbock, United States of America Natural Science Research Laboratory, Museum of Texas Tech University Lubbock United States of America; 2 Systematic Entomology Laboratory, ARS, USDA, c/o National Museum of Natural History, Smithsonian Institution, Washington, DC, United States of America Systematic Entomology Laboratory, ARS, USDA, c/o National Museum of Natural History, Smithsonian Institution Washington, DC United States of America

**Keywords:** Broad-nosed weevils, native species, morphotypes, morphological variation, host plants, distribution

## Abstract

**Background:**

The golden-headed weevil *Compsusauricephalus* is a native and fairly widespread species across the southern U.S.A. extending through Central America south to Panama. There are two recognised morphotypes of the species: the typical green form, with pink to cupreous head and part of the legs and the uniformly white to pale brown form. There are other Central and South American species of *Compsus* and related genera of similar appearance that make it challenging to provide accurate identifications of introduced species at ports of entry.

**New information:**

Here, we re-describe the species, provide images of the habitus, miscellaneous morphological structures and male and female genitalia. We discuss the morphological variation of *Compsusauricephalus* across its distributional range, by revising and updating its distributional range, based on data from entomological collections in the U.S.A. and Canada. The revised distribution of *C.auricephalus* extends as far south as Zacapa in Guatemala. Records south from there correspond to a different species, with affinities to *C.auricephalus* that we discuss and illustrate. We also discuss morphological affinities and differences with other similar species. Furthermore, we summarise information regarding the biology, host plants and natural enemies of *C.auricephalus*.

## Introduction

Broad-nosed weevils of the subfamily Entiminae Schönherr, 1823 (Schönherr 1823) are amongst the most diverse groups of beetles worldwide. Entimines are known for their polyphagous feeding habits (Marvaldi et al. 2014) and include some of the, arguably, most stunning and charismatic forms of weevils: the genus *Eupholus* Boisduval, 1835 (Boisduval 1835) (known as "smurf-weevils", see Van Dam et al. 2017), the genus *Pachyrhynchus* Germar, 1824 (Germar 1824; for example, Háva and Rukmane 2018) and the genus *Briarius* Fischer de Waldheim, 1829 (Fischer de Waldheim 1829) (formerly *Lamprocyphus* Marshall, 1922 (Marshall 1922) (see del Río and Lanteri 2003).

There are 55 tribes recognised within Entiminae (Alonso-Zarazaga and Lyal 1999), most of them distributed in particular biogeographic regions of the world (Marvaldi et al. 2014). The tribe Eustylini Lacordaire, 1863 (Lacordaire 1863) is restricted, for the most part, to the Neotropical region, grouping 23 genera and 334 described species (Franz 2012). Eustylines are amongst the most commonly found broad-nosed weevils in Northern South America (e.g. Girón and Cardona-Duque 2018) and include many relatively large (~7 to 25 mm) and strikingly coloured species. Some eustylines are known as serious agricultural pests, such as the infamous "diaprepres root weevil", *Diaprepesabbreviatus* (Linnaeus, 1758) (Linnaeus 1758), which attacks the roots and foliage of citrus, sugar cane, coffee and other cultivated plants throughout the Caribbean region and Florida (Hall 1995, Simpson et al. 1996, Weissling et al. 1998, Wolcott 1922). Only in Puerto Rico, *D.abbreviatus* feeds on the foliage of over 40 plant species (Martorell 1945). "It is estimated that *Diaprepesabbreviatus* causes about 70 million dollars in damage annually in Florida. Estimates show the weevil infests more than 100,000 acres of citrus" (Weissling et al. 1998).

Species identification of Eustylini is quite challenging, partly because of their diversity and the lack of revisionary (and comprehensive) taxonomic studies for the group, but also because of the high degree of overlapping characters amongst eustyline genera. Furthermore, the limits of Eustylini, as a tribe, are still not clearly defined, as some genera in the tribe Geonemini Gistel, 1848 (Gistel 1848) exhibit quite similar morphologies and have been shown to cluster together with eustylines in phylogenetic analyses (Franz 2012, Marvaldi et al. 2018). Indeed, recent studies in Eustylini have referred to the "*Exophthalmus* genus complex" (Franz 2012, Zhang and Franz 2015, Zhang et al. 2017), as a mainly Caribbean and Central American group, including the genera *Compsus* Schönherr, 1823 (Schönherr 1823), *Diaprepes* Schöenherr, 1823 (Schönherr 1823), *Eustylus* Schönherr, 1842 (Schönherr 1842), *Exophthalmus* Schönherr, 1823 (Schönherr 1823), *Exorides* Pascoe, 1881 (Pascoe 1881) (all Eustylini) and *Lachnopus* Schönherr, 1840 (Schönherr 1840) (Geonemini), amongst which, at certain points, the generic limits are indistinct. A similar situation occurs amongst the genera *Compsus*, *Brachyomus* Lacordaire, 1863 (Lacordaire 1863), *Chauliopleurus* Champion, 1911 (Champion 1911), *Exorides*, *Eustylus*, *Oxyderces* Schönherr, 1823 (Schönherr 1823) and *Xestogaster* Marshall, 1922 (Marshall 1922), here called the "*Compsus* genus complex", which includes nearly 180 species distributed almost exclusively throughout South America (O'Brien and Wibmer 1982, Wibmer and O'Brien 1986, Morrone 1999). The majority of species described in these genera remain only known from their original descriptions, have never been illustrated and only rarely those descriptions include information about the genitalia. Some geonemines, like *Epicaerus* Schönherr, 1834 (Schönherr 1834) and *Compsonomus* Jekel, 1875 (Jekel 1875), exhibit morphological similarities with members of the "*Compsus* genus complex".

The taxonomic confusion of the Eustylini became painfully evident with recent domestic and port interceptions in the U.S.A. of an unknown, colourful and eye-catching Colombian eustyline species loosely associated with imported cut flowers (L. Chamorro, pers. obs.). This eustyline species was tentatively identified in the taxonomically confused and diverse *Compsus* genus complex, but further identification required comparisons with type material deposited in European institutions.

**Figure 1.**
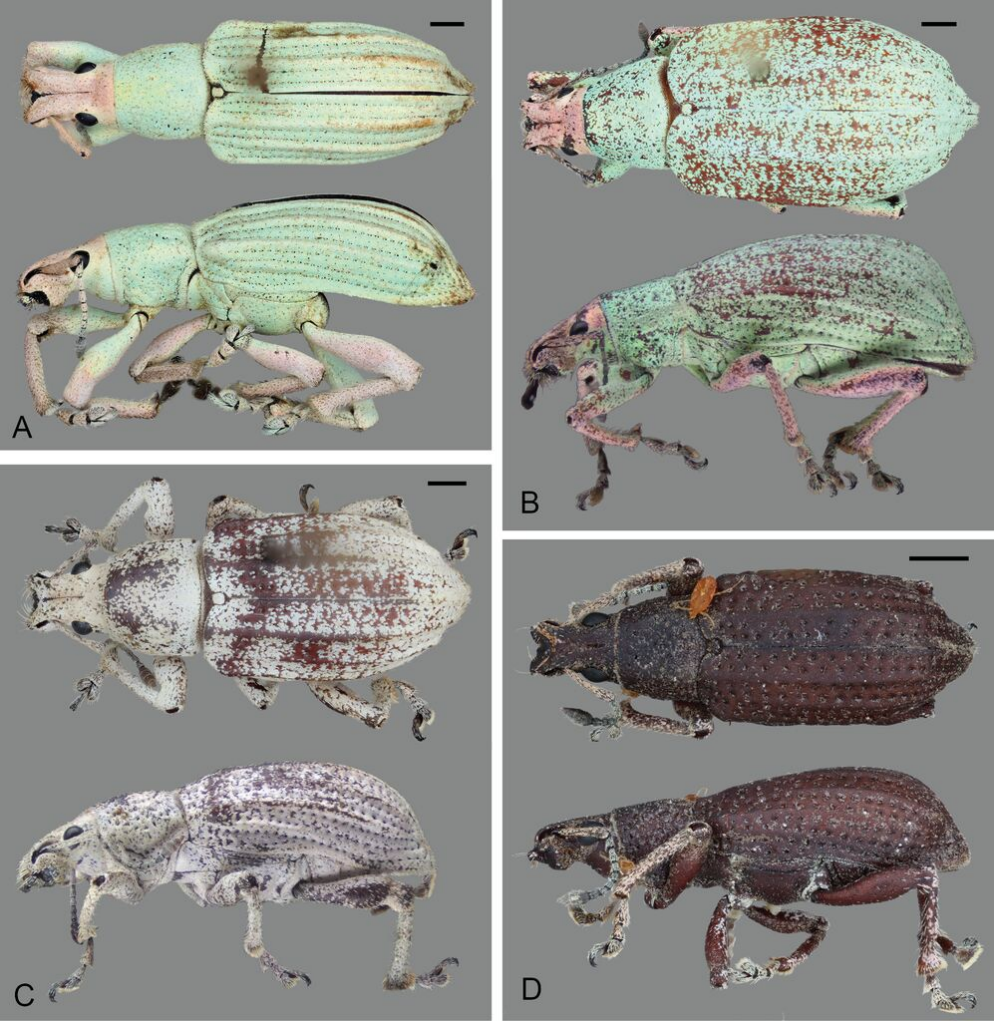
Habitus and variation of *Compsusauricephalus*: (A) Mexico, Veracruz (CWOC0047) dorsal, lateral, (B) Mississippi, Washington County (CWOC0045) dorsal, lateral, (C) Texas, Brooks County (CWOC0002) dorsal, lateral, (D) Mississippi, Washington County (CWOC0814) dorsal, lateral. Scale bars: 1 mm.

A single species in the *Compsus* genus complex occurs in the U.S.A., the golden-headed weevil, *Compsusauricephalus* (Say, 1824) (Say 1824) (Poole et al. 1996). This species ranges as far north as Illinois, west to Colorado and Arizona, east to Georgia and throughout Central America as far south as Panama (Wibmer and O'Brien 1986, Morrone 1999); in addition, it has been intercepted at ports in Ontario, Canada (McNamara 1991). Within this range, *C.auricephalus* exhibits two main colour morphs: predominantly green with pink/coppery head and part of the legs or completely white to pale brown (see Fig. 1). Champion (1911) highlighted that specimens from southern populations (e.g. Mexico (Acapulco), Guatemala, Costa Rica, Nicaragua, Panama) had a more elongate body, produced and acuminate elytral apices and strongly costate alternating interstriae. In addition to variation in colour, the species exhibits broad size variation (8–15 mm), with some males being half the size of the females.

Even though *C.auricephalus* can be regarded as a highly variable species, being the only species of the genus present in the U.S.A. and recognising its variation is probably good enough to differentiate it from other U.S.A. entimines. The problem is that the genus currently has over a hundred species across its distributional range, with some of them looking quite similar to either or both of the colour morphs of *C.auricephalus*. This situation may lead to misidentifications, especially of Central American specimens, causing problems at ports of entry given the current inability of determining if intercepted specimens belong to a native or exotic species.

This study aims to clarify the status of *Compsusauricephalus* by (1) presenting a full re-description of the species with images representing its morphological characteristics and range of variation, (2) comparing *C.auricephalus* to externally similar species, (3) updating distributional records including a map and (4) presenting a summary of biological information including a list of host plants and natural enemies for the species, with a discussion on the status of *C.auricephalus* as a crop pest. The mouthparts, hind wings and male and female genitalia are described here for the first time.

## Materials and methods

### Specimen data

Over 700 specimens were directly examined (Suppl. material 1 in addition to specimens listed under Materials below). Type material of miscellaneous species in the *Compsus* genus complex were examined in order to differentiate *C.auricephalus* from similarly-looking species. Examined specimens are deposited at the following institutions:

- **ASUCOB** Charles W. O'Brien Collection, Arizona State University, Tempe, Arizona (Arizona State University Biocollections 2019; Charles O’Brien, Emmy Engaser, Sangmi Lee, Nico Franz).
- **CMNC** Canadian Museum of Nature, Ottawa, Canada (Robert Anderson).
- **MNHN** Muséum National d’Histoire Naturelle, Paris, France (Hélène Perrin and Antoine Mantilleri).
- **NHMUK** The Natural History Museum [formerly British Museum (Natural History)], London, United Kingdom (Maxwell Barclay).
- **NHRS** Naturhistoriska Riksmuseet, Stockholm, Sweden (Johannes Bergsten).
- **OSUC** C.A. Triplehorn Insect Collection, Ohio State University, Columbus, Ohio (Johnson and Cora 2018; Luciana Musetti and Norman Johnson).
- **TAMUIC** Texas A & M University, College Station, Texas (Texas A&M University Insect Collection 2020; Karen Wright).
- **TTUZ** Texas Tech University, Lubbock, Texas (Museum of Texas Tech University (TTU) 2020; Jennifer Girón).
- **USNM** U.S. National Museum of Natural History, Smithsonian Institution, Washington, DC. (Lourdes Chamorro and Floyd Shockley).
- **ZMUK** Zoological Museum of Kiel University, Kiel, Germany (Michael Kuhlmann) loan initiated by **ZMUC** Natural History Museum of Denmark (University of Copenhagen, Zoological Museum, Copenhagen, Denmark (Alexey Solodovnikov).

Data for ASUCOB (part), CMNC, NHMUK and USNM were compiled into a DarwinCore file (Suppl. material 1). In addition, preserved specimen data were retrieved via SCAN (SCAN 2020) from the following collections:

- **ASUHIC** Hasbrouck Insect Collection, Arizona State University, Tempe, Arizona (Arizona State University Biocollections 2020).
- **AUEM** Auburn University Museum of Natural History Entomology, Auburn University, Auburn, Alabama (Callahan 2019).
- **CASM** Museum of Natural History, Chicago Academy of Sciences, Chicago, Illinois (Roberts 2020).
- **MSUC** Michigan State University, East Lansing, Michigan (A.J. Cook Arthropod Research Collection 2020).
- **UAIC** University of Arizona Insect Collection, University of Arizona, Tucson, Arizona (University of Arizona Insect Collection 2020).
- **UCFC** Stuart M. Fullerton Collection of Arthropods, University of Central Florida, Orlando, Florida (Song and Johnson 2018).
- **UMNH** Utah Museum of Natural History, University of Utah, Salt Lake City, Utah (Natural History Museum of Utah (UMNH) 2020).

These datasets were also recovered through GBIF (GBIF.org 2020). Additional GBIF records (GBIF.org 2020) were recovered from the following datasets:

- Base de datos de la Colección zoológica del Instituto de Investigación de zonas desérticas de la Universidad Autónoma de San Luis Potosí (BDCZIID-UASLP) (Martínez de la Vega G and Comisión nacional para el conocimiento y uso de la biodiversidad 2018).
- Captura de datos de la Colección de Curculionoidea (Insecta: Coleoptera) de la Universidad Autónoma de Querétaro (Jones RW and Comisión nacional para el conocimiento y uso de la biodiversidad 2018).
- Computarización de la Colección Nacional de insectos Dr. Alfredo Barrera Marín del Museo de Historia Natural de la Ciudad de México (Díaz Batres ME and Comisión nacional para el conocimiento y uso de la biodiversidad 2018).
- Computarización de la colección científica del proyecto de control biológico de malezas de CSIRO-Australia (Segura Ponce de León 2020Segura Ponce de León, R and Comisión nacional para el conocimiento y uso de la biodiversidad C 2020).
- Elaboración de la base de datos de los ejemplares de la colección general de insectos adultos de la Dirección General de Sanidad Vegetal (Vega Ortiz, H E and Comisión nacional para el conocimiento y uso de la biodiversidad C 2020).

The dataset was recovered from GBIF on 5 June 2020, contains 728 occurrence records and can be downloaded from https://doi.org/10.15468/dl.rat633. 'Human observations' from iNaturalist (iNaturalist.org 2020; as opposed to 'preserved specimens', see Darwin Core terminology http://rs.tdwg.org/dwc/terms.htm) and Bugguide (VanDyk et al. 2020), as recovered from GBIF, were verified and included in our dataset, representing 124 (17%) of the GBIF records. Only two records, both based on human observations, (Oak Mountain State Park, Shelby County, Alabama [https://bugguide.net/node/view/1694325] and Fondes Amandes Rd, Port of Spain, Trinidad and Tobago [https://www.inaturalist.org/observations/32103921]) were excluded given that the identity of the specimens cannot be confirmed from the pictures provided.

For the following collections, specimen data were obtained directly from collection’s curators and compiled into our DarwinCore file (Suppl. material 1):

- **CLEV** Cleveland Museum of Natural History, Columbus, Ohio (Nicole Gunter).
- **CMNC** Canadian Museum of Nature, Ottawa, Canada (Robert Anderson).
- **INHS** Illinois Natural History Survey, Champaign, Illinois (Tommy McElrath, Dmitriev 2015).
- **LSAM** Louisiana State Arthropod Museum, Louisiana State University, Baton Rouge, Louisiana (Victoria Bayless, Nathan Lord).
- **MEM** Mississippi State University, Mississippi, Mississippi (Terence Schiefer).

In total, 1606 specimen records are included in this study. Part of the coordinates presented in Suppl. material 1 (also available at https://doi.org/10.15468/chpymx) were obtained by approximating the locality data via Google Maps (https://www.google.com/maps). The distribution map was created using SimpleMappr (Shorthouse 2010). Collection codes, listed here, correspond to those listed in Evenhuis 2020. Information on host plants was obtained both from literature records and label data.

### Morphological methods

Specimens were examined using an AmScope SM-1TSZZ-144S stereomicroscope (magnification: 3.5X-180X) and a Zeiss Discovery v8. Genitalia dissections were prepared by removing the entire abdomen from the specimen and opening it along one side; then the abdomen was submerged in a solution of 10% potassium hydroxide (KOH) and heated to 50°C overnight. Afterwards, the macerated abdomen was submerged in glacial acetic acid for 10 minutes and then rinsed with distilled water. Dissections were ultimately performed by placing the cleared abdomen on a microscope slide with a drop of glycerine.

Habitus photographs were taken with a Visionary Digital Passport II imaging system (Visionary Digital, Los Angeles, CA), using a Canon MP-E 65mm lens f/1:2.8 1–5X macro lens mounted on a Canon 5D Mark III camera body and the Macropod Pro 3D system using Canon MP-E 65mm lens f/2.8 1-5x macro lens on a Canon 6D camera body on Focus stacking rails controlled by StackShot and Canon MT-24EX Macro Twin Lite flash units (Macroscopic Solutions, Connecticut, USA). Images of internal structures were produced by stacking images taken through a Canon EOS 5D Mark II camera attached to an AmScope SM-1TSZZ-144S stereomicroscope or a Nikon Optiphot microscope at 100× magnification. The serial images were processed using Helicon Focus 5.3 software (Helicon Soft Ltd 2013) and Zerene Stacker (Zerene Systems LLC 2019).

The species re-description follows Franz (2010), for the most part and is based on a series of specimens examined. Morphological terms are in agreement with Torre-Bueno (Nichols 1989) and Lawrence and Ślipiński (2013), with additional terms for structures of the mouthparts (Ting 1936), metendosternite (Velázquez de Castro 1998), wings (Zherikhin and Gratshev 1995) and the male (Wanat 2007, Boudinot 2018, Génier 2019) and female genitalia (Howden 1995, Gaiger and Vanin 2008).

## Taxon treatments

### Compsus auricephalus

(Say, 1824)

E064A6FB-2431-5C9F-9AC5-43B30320C6C0

https://www.biodiversitylibrary.org/page/24668962

https://www.gbif.org/species/5015904

Compsus auricephalus **Taxonomy**
Compsus auricephalus CurculionidaeLatreille 1802
Compsus auricephalus EntiminaeSchönherr 1823
Compsus auricephalus EustyliniLacordaire 1863
Compsus auricephalus *Compsus*Schönherr 1823
Compsus auricephalus *Curculioelegans*Olivier 1807*Curculioargyreus*Linnaeus 1758
Compsus auricephalus ***Compsusauricephalus* (Say, 1824)**
Curculio auricephalus Say, 1824: 310 (Say 1824)
Platyomus auricephalus (Boheman 1833: 645) (new combination in Schönherr 1833)
Platyomus auriceps Schönherr, 1840: 183 (Schönherr 1840, Fig. 2)
Compsus auricephalus *C.auricephalus*[Bibr B5732871][Bibr B5732861]*Platyomusauricephalus*Schönherr 1833*Platyomusauriceps*[Bibr B5732464][Bibr B5732774][Bibr B5732603]*C.auricephalusC.isabellinus*Schönherr 1833*C.auricephalus*Blatchley and Leng (1916)Pierce (1916)

#### Materials

- **Type status:** Other material. **Occurrence:** occurrenceDetails: http://api.gbif.org/v1/occurrence/search?occurrenceId=6cc2275b-b2cd-4ce8-ad3e-dec883a2ed72; catalogNumber: ASUHIC0019442; recordedBy: D.A. Rider; individualCount: 1; occurrenceID: 6cc2275b-b2cd-4ce8-ad3e-dec883a2ed72; **Taxon:** taxonID: 8801; scientificName: Compsusauricephalus Say, 1824; kingdom: Animalia; phylum: Arthropoda; class: Insecta; order: Coleoptera; family: Curculionidae; genus: Compsus; specificEpithet: auricephalus; taxonRank: SPECIES; taxonomicStatus: ACCEPTED; **Location:** country: United States of America; countryCode: US; stateProvince: Louisiana; county: Saint Landry Parish; locality: Thistlethwaite National Wildlife Refuge; decimalLatitude: 30.669309; decimalLongitude: -92.022851; geodeticDatum: WGS84; georeferencedBy: Andrew Jansen; georeferenceSources: GeoLocate; georeferenceVerificationStatus: requires verification; **Identification:** identifiedBy: Charles W. O'Brien; dateIdentified: 2011-01-01T00:00:00; **Event:** eventDate: 1989-05-07T00:00:00; startDayOfYear: 127; year: 1989; month: 5; day: 7; **Record Level:** modified: 2019-06-07T10:46:31.000+0000; rights: http://creativecommons.org/publicdomain/zero/1.0/; collectionID: 98d9b8ed-08d6-47fc-b324-2853e44d75d1; institutionCode: ASU; collectionCode: ASUHIC; basisOfRecord: PRESERVED_SPECIMEN
- **Type status:** Other material. **Occurrence:** occurrenceDetails: http://api.gbif.org/v1/occurrence/search?occurrenceId=b1bb5a9c-1a79-4fc8-b570-e7848a1fd401; catalogNumber: ASUHIC0019443; recordedBy: E.G. Riley; individualCount: 1; occurrenceID: b1bb5a9c-1a79-4fc8-b570-e7848a1fd401; **Taxon:** taxonID: 8801; scientificName: Compsusauricephalus Say, 1824; kingdom: Animalia; phylum: Arthropoda; class: Insecta; order: Coleoptera; family: Curculionidae; genus: Compsus; specificEpithet: auricephalus; taxonRank: SPECIES; taxonomicStatus: ACCEPTED; **Location:** country: United States of America; countryCode: US; stateProvince: Texas; county: Cameron County; locality: Sabal Palm Grove Sanctuary; decimalLatitude: 25.8525; decimalLongitude: -97.4175; geodeticDatum: WGS84; georeferencedBy: Andrew Jansen; georeferenceSources: SCAN; georeferenceVerificationStatus: requires verification; **Identification:** identifiedBy: Charles W. O'Brien; dateIdentified: 2006-01-01T00:00:00; **Event:** eventDate: 1986-03-28T00:00:00; startDayOfYear: 87; year: 1986; month: 3; day: 28; **Record Level:** modified: 2019-06-07T10:46:31.000+0000; rights: http://creativecommons.org/publicdomain/zero/1.0/; collectionID: 98d9b8ed-08d6-47fc-b324-2853e44d75d1; institutionCode: ASU; collectionCode: ASUHIC; basisOfRecord: PRESERVED_SPECIMEN
- **Type status:** Other material. **Occurrence:** occurrenceDetails: http://api.gbif.org/v1/occurrence/search?occurrenceId=d1a02bff-93a7-4746-8a40-829ac1a4e359; catalogNumber: TTU-Z_048376; recordedBy: Bob Starkey; individualCount: 1; lifeStage: ADULT; preparations: dry pinned; otherCatalogNumbers: TTU1997-058; occurrenceID: d1a02bff-93a7-4746-8a40-829ac1a4e359; **Taxon:** taxonID: 8801; scientificName: Compsusauricephalus Say, 1824; kingdom: Animalia; phylum: Arthropoda; class: Insecta; order: Coleoptera; family: Curculionidae; genus: Compsus; specificEpithet: auricephalus; taxonRank: SPECIES; taxonomicStatus: ACCEPTED; **Location:** country: United States of America; countryCode: US; stateProvince: Texas; county: Lubbock; locality: 2 Mi. N of Lubbock; decimalLatitude: 33.66685; decimalLongitude: -101.83692; geodeticDatum: WGS84; coordinateUncertaintyInMeters: 1891; **Identification:** identifiedBy: J. Girón; dateIdentified: 2020-01-01T00:00:00; **Event:** eventDate: 1980-06-06T00:00:00; startDayOfYear: 158; year: 1980; month: 6; day: 6; **Record Level:** modified: 2015-01-08T00:00:00.000+0000; rights: http://creativecommons.org/licenses/by-nc/3.0/; rightsHolder: Museum of Texas Tech University; accessRights: CC BY-NC (Attribution-Non-Commercial); collectionID: d4e788b4-5d52-47a3-873e-227c6df49c7b; institutionCode: TTU; collectionCode: TTU-Z; basisOfRecord: PRESERVED_SPECIMEN
- **Type status:** Other material. **Occurrence:** occurrenceDetails: http://api.gbif.org/v1/occurrence/search?occurrenceId=4832064b-a8ab-4fc5-b475-c15a59db588e; catalogNumber: TTU-Z_213647; recordNumber: 13647; recordedBy: Ward; Brothers; individualCount: 1; otherCatalogNumbers: TTU1997-058; occurrenceID: 4832064b-a8ab-4fc5-b475-c15a59db588e; **Taxon:** taxonID: 8801; scientificName: Compsusauricephalus Say, 1824; kingdom: Animalia; phylum: Arthropoda; class: Insecta; order: Coleoptera; family: Curculionidae; genus: Compsus; specificEpithet: auricephalus; taxonRank: SPECIES; taxonomicStatus: ACCEPTED; **Location:** country: Mexico; countryCode: MX; stateProvince: Tamaulipas; locality: 3 miles North of Cuidad Victoria; verbatimElevation: 800; decimalLatitude: 31.044186; decimalLongitude: -112.103; geodeticDatum: WGS84; coordinateUncertaintyInMeters: 6954; georeferencedBy: Alex Gregg (2014-08-14 13:58:34); georeferenceSources: georef batch tool 2014-08-14; GeoLocate; georeferenceVerificationStatus: reviewed - high confidence; **Identification:** identifiedBy: C.W. O'Brien; **Event:** eventDate: 1971-06-24T00:00:00; startDayOfYear: 175; year: 1971; month: 6; day: 24; verbatimEventDate: VI-24-1971; **Record Level:** modified: 2015-01-08T00:00:00.000+0000; rights: http://creativecommons.org/licenses/by-nc/3.0/; rightsHolder: Museum of Texas Tech University; accessRights: CC BY-NC (Attribution-Non-Commercial); collectionID: d4e788b4-5d52-47a3-873e-227c6df49c7b; institutionCode: TTU; collectionCode: TTU-Z; basisOfRecord: PRESERVED_SPECIMEN
- **Type status:** Other material. **Occurrence:** occurrenceDetails: http://api.gbif.org/v1/occurrence/search?occurrenceId=806e4d86-48ad-48d4-af87-67227271b55a; catalogNumber: TTU-Z_219309; recordedBy: R. R. Rogers; individualCount: 1; preparations: pinned; otherCatalogNumbers: TTU1997-058; occurrenceID: 806e4d86-48ad-48d4-af87-67227271b55a; **Taxon:** taxonID: 8801; scientificName: Compsusauricephalus Say, 1824; kingdom: Animalia; phylum: Arthropoda; class: Insecta; order: Coleoptera; family: Curculionidae; genus: Compsus; specificEpithet: auricephalus; taxonRank: SPECIES; taxonomicStatus: ACCEPTED; **Location:** country: United States of America; countryCode: US; stateProvince: Texas; county: Starr; decimalLatitude: 26.562153; decimalLongitude: -98.7384; geodeticDatum: WGS84; coordinateUncertaintyInMeters: 53416; georeferenceRemarks: 88 High STARR COUNTY; **Identification:** identifiedBy: D. R. Whitehead; **Event:** eventDate: 1968-04-09T00:00:00; startDayOfYear: 100; year: 1968; month: 4; day: 9; verbatimEventDate: 9 IV 1968; habitat: Prosopisglandulosa; **Record Level:** modified: 2015-01-08T00:00:00.000+0000; rights: http://creativecommons.org/licenses/by-nc/3.0/; rightsHolder: Museum of Texas Tech University; accessRights: CC BY-NC (Attribution-Non-Commercial); collectionID: d4e788b4-5d52-47a3-873e-227c6df49c7b; institutionCode: TTU; collectionCode: TTU-Z; basisOfRecord: PRESERVED_SPECIMEN
- **Type status:** Other material. **Occurrence:** occurrenceDetails: http://api.gbif.org/v1/occurrence/search?occurrenceId=6367d96d-1c14-488c-baf5-51c318d77b1a; catalogNumber: TTU-Z_219302; recordedBy: D. D. Collins; individualCount: 1; preparations: pinned; otherCatalogNumbers: TTU1997-058; occurrenceID: 6367d96d-1c14-488c-baf5-51c318d77b1a; **Taxon:** taxonID: 8801; scientificName: Compsusauricephalus Say, 1824; kingdom: Animalia; phylum: Arthropoda; class: Insecta; order: Coleoptera; family: Curculionidae; genus: Compsus; specificEpithet: auricephalus; taxonRank: SPECIES; taxonomicStatus: ACCEPTED; **Location:** country: United States of America; countryCode: US; stateProvince: Texas; county: Hall; locality: Estelline; decimalLatitude: 34.54667; decimalLongitude: -100.43778; geodeticDatum: WGS84; coordinateUncertaintyInMeters: 1285; georeferenceSources: GeoLocate; **Identification:** identifiedBy: C. W. O'Brien; dateIdentified: 1978-01-01T00:00:00; **Event:** eventDate: 1968-06-10T00:00:00; startDayOfYear: 162; year: 1968; month: 6; day: 10; verbatimEventDate: 6/10/1968; **Record Level:** modified: 2015-01-08T00:00:00.000+0000; rights: http://creativecommons.org/licenses/by-nc/3.0/; rightsHolder: Museum of Texas Tech University; accessRights: CC BY-NC (Attribution-Non-Commercial); collectionID: d4e788b4-5d52-47a3-873e-227c6df49c7b; institutionCode: TTU; collectionCode: TTU-Z; basisOfRecord: PRESERVED_SPECIMEN
- **Type status:** Other material. **Occurrence:** occurrenceDetails: http://api.gbif.org/v1/occurrence/search?occurrenceId=4712568f-cd4c-44b3-8709-e1f7f12ffd69; catalogNumber: TTU-Z_219303; recordedBy: D. D. Collins; individualCount: 1; preparations: pinned; otherCatalogNumbers: TTU1997-058; occurrenceID: 4712568f-cd4c-44b3-8709-e1f7f12ffd69; **Taxon:** taxonID: 8801; scientificName: Compsusauricephalus Say, 1824; kingdom: Animalia; phylum: Arthropoda; class: Insecta; order: Coleoptera; family: Curculionidae; genus: Compsus; specificEpithet: auricephalus; taxonRank: SPECIES; taxonomicStatus: ACCEPTED; **Location:** country: United States of America; countryCode: US; stateProvince: Texas; county: Hall; locality: Estelline; decimalLatitude: 34.54667; decimalLongitude: -100.43778; geodeticDatum: WGS84; coordinateUncertaintyInMeters: 1285; georeferenceSources: GeoLocate; **Identification:** identifiedBy: C.W. O'Brien; **Event:** eventDate: 1968-06-10T00:00:00; startDayOfYear: 162; year: 1968; month: 6; day: 10; verbatimEventDate: 6/10/1968; **Record Level:** modified: 2015-01-08T00:00:00.000+0000; rights: http://creativecommons.org/licenses/by-nc/3.0/; rightsHolder: Museum of Texas Tech University; accessRights: CC BY-NC (Attribution-Non-Commercial); collectionID: d4e788b4-5d52-47a3-873e-227c6df49c7b; institutionCode: TTU; collectionCode: TTU-Z; basisOfRecord: PRESERVED_SPECIMEN
- **Type status:** Other material. **Occurrence:** occurrenceDetails: http://api.gbif.org/v1/occurrence/search?occurrenceId=90809cdc-23cb-426a-b5b9-be2f6e0c7e09; catalogNumber: TTU-Z_219304; recordedBy: D. D. Collins; individualCount: 1; preparations: pinned; otherCatalogNumbers: TTU1997-058; occurrenceID: 90809cdc-23cb-426a-b5b9-be2f6e0c7e09; **Taxon:** taxonID: 8801; scientificName: Compsusauricephalus Say, 1824; kingdom: Animalia; phylum: Arthropoda; class: Insecta; order: Coleoptera; family: Curculionidae; genus: Compsus; specificEpithet: auricephalus; taxonRank: SPECIES; taxonomicStatus: ACCEPTED; **Location:** country: United States of America; countryCode: US; stateProvince: Texas; county: Hall; locality: Estelline; decimalLatitude: 34.54667; decimalLongitude: -100.43778; geodeticDatum: WGS84; coordinateUncertaintyInMeters: 1285; georeferenceSources: GeoLocate; **Identification:** identifiedBy: C.W. O'Brien; dateIdentified: 1970-01-01T00:00:00; **Event:** eventDate: 1968-06-10T00:00:00; startDayOfYear: 162; year: 1968; month: 6; day: 10; verbatimEventDate: 6/10/1968; **Record Level:** modified: 2015-01-08T00:00:00.000+0000; rights: http://creativecommons.org/licenses/by-nc/3.0/; rightsHolder: Museum of Texas Tech University; accessRights: CC BY-NC (Attribution-Non-Commercial); collectionID: d4e788b4-5d52-47a3-873e-227c6df49c7b; institutionCode: TTU; collectionCode: TTU-Z; basisOfRecord: PRESERVED_SPECIMEN
- **Type status:** Other material. **Occurrence:** occurrenceDetails: http://api.gbif.org/v1/occurrence/search?occurrenceId=a3d0f53f-5124-44c8-9e64-91f68b54daa6; catalogNumber: TTU-Z_219305; recordedBy: D. D. Collins; individualCount: 1; preparations: pinned; otherCatalogNumbers: TTU1997-058; occurrenceID: a3d0f53f-5124-44c8-9e64-91f68b54daa6; **Taxon:** taxonID: 8801; scientificName: Compsusauricephalus Say, 1824; kingdom: Animalia; phylum: Arthropoda; class: Insecta; order: Coleoptera; family: Curculionidae; genus: Compsus; specificEpithet: auricephalus; taxonRank: SPECIES; taxonomicStatus: ACCEPTED; **Location:** country: United States of America; countryCode: US; stateProvince: Texas; county: Hall; locality: Estelline; decimalLatitude: 34.54667; decimalLongitude: -100.43778; geodeticDatum: WGS84; coordinateUncertaintyInMeters: 1285; georeferenceSources: GeoLocate; **Identification:** identifiedBy: C.W. O'Brien; dateIdentified: 1970-01-01T00:00:00; **Event:** eventDate: 1968-06-10T00:00:00; startDayOfYear: 162; year: 1968; month: 6; day: 10; verbatimEventDate: 6/10/1968; **Record Level:** modified: 2015-01-08T00:00:00.000+0000; rights: http://creativecommons.org/licenses/by-nc/3.0/; rightsHolder: Museum of Texas Tech University; accessRights: CC BY-NC (Attribution-Non-Commercial); collectionID: d4e788b4-5d52-47a3-873e-227c6df49c7b; institutionCode: TTU; collectionCode: TTU-Z; basisOfRecord: PRESERVED_SPECIMEN
- **Type status:** Other material. **Occurrence:** occurrenceDetails: http://api.gbif.org/v1/occurrence/search?occurrenceId=a5bdd73f-4d99-4222-90e6-7290a1dda179; catalogNumber: TTU-Z_219306; recordedBy: D. D. Collins; individualCount: 1; lifeStage: ADULT; preparations: pinned; otherCatalogNumbers: TTU1997-058; occurrenceID: a5bdd73f-4d99-4222-90e6-7290a1dda179; **Taxon:** taxonID: 8801; scientificName: Compsusauricephalus Say, 1824; kingdom: Animalia; phylum: Arthropoda; class: Insecta; order: Coleoptera; family: Curculionidae; genus: Compsus; specificEpithet: auricephalus; taxonRank: SPECIES; taxonomicStatus: ACCEPTED; **Location:** country: United States of America; countryCode: US; stateProvince: Texas; county: Hall; decimalLatitude: 34.54667; decimalLongitude: -100.43778; geodeticDatum: WGS84; coordinateUncertaintyInMeters: 1285; georeferenceSources: GeoLocate; **Identification:** identifiedBy: C.W. O'Brien; dateIdentified: 1970-01-01T00:00:00; **Event:** eventDate: 1968-06-10T00:00:00; startDayOfYear: 162; year: 1968; month: 6; day: 10; verbatimEventDate: 6/10/1968; **Record Level:** modified: 2015-01-08T00:00:00.000+0000; rights: http://creativecommons.org/licenses/by-nc/3.0/; rightsHolder: Museum of Texas Tech University; accessRights: CC BY-NC (Attribution-Non-Commercial); collectionID: d4e788b4-5d52-47a3-873e-227c6df49c7b; institutionCode: TTU; collectionCode: TTU-Z; basisOfRecord: PRESERVED_SPECIMEN
- **Type status:** Other material. **Occurrence:** occurrenceDetails: http://api.gbif.org/v1/occurrence/search?occurrenceId=b53e7fa2-baf4-4a03-a3ca-769c3ded1023; catalogNumber: TTU-Z_219307; recordedBy: D. D. Collins; individualCount: 1; preparations: pinned; otherCatalogNumbers: TTU1997-058; occurrenceID: b53e7fa2-baf4-4a03-a3ca-769c3ded1023; **Taxon:** taxonID: 8801; scientificName: Compsusauricephalus Say, 1824; kingdom: Animalia; phylum: Arthropoda; class: Insecta; order: Coleoptera; family: Curculionidae; genus: Compsus; specificEpithet: auricephalus; taxonRank: SPECIES; taxonomicStatus: ACCEPTED; **Location:** country: United States of America; countryCode: US; stateProvince: Texas; county: Hall; locality: Estelline; decimalLatitude: 34.54667; decimalLongitude: -100.43778; geodeticDatum: WGS84; coordinateUncertaintyInMeters: 1285; georeferenceSources: GeoLocate; **Identification:** identifiedBy: C.W. O'Brien; dateIdentified: 1970-01-01T00:00:00; **Event:** eventDate: 1968-06-10T00:00:00; startDayOfYear: 162; year: 1968; month: 6; day: 10; verbatimEventDate: 6/10/1968; **Record Level:** modified: 2015-01-08T00:00:00.000+0000; rights: http://creativecommons.org/licenses/by-nc/3.0/; rightsHolder: Museum of Texas Tech University; accessRights: CC BY-NC (Attribution-Non-Commercial); collectionID: d4e788b4-5d52-47a3-873e-227c6df49c7b; institutionCode: TTU; collectionCode: TTU-Z; basisOfRecord: PRESERVED_SPECIMEN
- **Type status:** Other material. **Occurrence:** occurrenceDetails: http://api.gbif.org/v1/occurrence/search?occurrenceId=308ccd72-428a-4119-aeb2-71cb9f4052e3; catalogNumber: TTU-Z_219297; occurrenceRemarks: S - 108 - F; recordedBy: Wayne H. Swenson; individualCount: 1; preparations: pinned; otherCatalogNumbers: TTU1997-058; occurrenceID: 308ccd72-428a-4119-aeb2-71cb9f4052e3; **Taxon:** taxonID: 8801; scientificName: Compsusauricephalus Say, 1824; kingdom: Animalia; phylum: Arthropoda; class: Insecta; order: Coleoptera; family: Curculionidae; genus: Compsus; specificEpithet: auricephalus; taxonRank: SPECIES; taxonomicStatus: ACCEPTED; **Location:** country: United States of America; countryCode: US; stateProvince: Texas; county: King; locality: Guthrie; decimalLatitude: 33.62056; decimalLongitude: -100.3225; geodeticDatum: WGS84; coordinateUncertaintyInMeters: 3036; georeferenceSources: GeoLocate; **Identification:** identifiedBy: R. E. Warner; **Event:** eventDate: 1968-06-18T00:00:00; startDayOfYear: 170; year: 1968; month: 6; day: 18; verbatimEventDate: 18-19 June 1968; **Record Level:** modified: 2015-01-08T00:00:00.000+0000; rights: http://creativecommons.org/licenses/by-nc/3.0/; rightsHolder: Museum of Texas Tech University; accessRights: CC BY-NC (Attribution-Non-Commercial); collectionID: d4e788b4-5d52-47a3-873e-227c6df49c7b; institutionCode: TTU; collectionCode: TTU-Z; basisOfRecord: PRESERVED_SPECIMEN
- **Type status:** Other material. **Occurrence:** occurrenceDetails: http://api.gbif.org/v1/occurrence/search?occurrenceId=42222edb-506a-413b-9b0f-ae59c8c38c93; catalogNumber: TTU-Z_219298; recordedBy: Wayne H. Swenson; individualCount: 1; preparations: pinned; otherCatalogNumbers: TTU1997-058; occurrenceID: 42222edb-506a-413b-9b0f-ae59c8c38c93; **Taxon:** taxonID: 8801; scientificName: Compsusauricephalus Say, 1824; kingdom: Animalia; phylum: Arthropoda; class: Insecta; order: Coleoptera; family: Curculionidae; genus: Compsus; specificEpithet: auricephalus; taxonRank: SPECIES; taxonomicStatus: ACCEPTED; **Location:** country: United States of America; countryCode: US; stateProvince: Texas; county: King; locality: Guthrie; decimalLatitude: 33.62056; decimalLongitude: -100.3225; geodeticDatum: WGS84; coordinateUncertaintyInMeters: 3036; georeferenceSources: GeoLocate; **Identification:** identifiedBy: R. E. Warner; **Event:** eventDate: 1968-06-07T00:00:00; startDayOfYear: 159; year: 1968; month: 6; day: 7; verbatimEventDate: 7-Jun-68; **Record Level:** modified: 2014-07-25T00:00:00.000+0000; rights: http://creativecommons.org/licenses/by-nc/3.0/; rightsHolder: Museum of Texas Tech University; accessRights: CC BY-NC (Attribution-Non-Commercial); collectionID: d4e788b4-5d52-47a3-873e-227c6df49c7b; institutionCode: TTU; collectionCode: TTU-Z; basisOfRecord: PRESERVED_SPECIMEN
- **Type status:** Other material. **Occurrence:** occurrenceDetails: http://api.gbif.org/v1/occurrence/search?occurrenceId=cf7b5d95-e819-4092-84d0-355e0fbd132c; catalogNumber: TTU-Z_219299; occurrenceRemarks: S - 108 - F; recordedBy: Wayne H. Swenson; individualCount: 1; preparations: pinned; otherCatalogNumbers: TTU1997-058; occurrenceID: cf7b5d95-e819-4092-84d0-355e0fbd132c; **Taxon:** taxonID: 8801; scientificName: Compsusauricephalus Say, 1824; kingdom: Animalia; phylum: Arthropoda; class: Insecta; order: Coleoptera; family: Curculionidae; genus: Compsus; specificEpithet: auricephalus; taxonRank: SPECIES; taxonomicStatus: ACCEPTED; **Location:** country: United States of America; countryCode: US; stateProvince: Texas; county: King; locality: Guthrie; decimalLatitude: 33.62056; decimalLongitude: -100.3225; geodeticDatum: WGS84; coordinateUncertaintyInMeters: 3036; georeferenceSources: GeoLocate; **Identification:** identifiedBy: R. E. Warner; **Event:** eventDate: 1968-06-18T00:00:00; startDayOfYear: 170; year: 1968; month: 6; day: 18; verbatimEventDate: 18-19 June 1968; **Record Level:** modified: 2015-01-08T00:00:00.000+0000; rights: http://creativecommons.org/licenses/by-nc/3.0/; rightsHolder: Museum of Texas Tech University; accessRights: CC BY-NC (Attribution-Non-Commercial); collectionID: d4e788b4-5d52-47a3-873e-227c6df49c7b; institutionCode: TTU; collectionCode: TTU-Z; basisOfRecord: PRESERVED_SPECIMEN
- **Type status:** Other material. **Occurrence:** occurrenceDetails: http://api.gbif.org/v1/occurrence/search?occurrenceId=b0f2136f-ac11-4e38-affd-cdb2e898bc06; catalogNumber: TTU-Z_219300; occurrenceRemarks: S - 108 - F; recordedBy: Wayne H. Swenson; individualCount: 1; preparations: pinned; otherCatalogNumbers: TTU1997-058; occurrenceID: b0f2136f-ac11-4e38-affd-cdb2e898bc06; **Taxon:** taxonID: 8801; scientificName: Compsusauricephalus Say, 1824; kingdom: Animalia; phylum: Arthropoda; class: Insecta; order: Coleoptera; family: Curculionidae; genus: Compsus; specificEpithet: auricephalus; taxonRank: SPECIES; taxonomicStatus: ACCEPTED; **Location:** country: United States of America; countryCode: US; stateProvince: Texas; county: King; locality: Guthrie; decimalLatitude: 33.62056; decimalLongitude: -100.3225; geodeticDatum: WGS84; coordinateUncertaintyInMeters: 3036; georeferenceSources: GeoLocate; **Identification:** identifiedBy: R. E. Warner; **Event:** eventDate: 1968-06-28T00:00:00; startDayOfYear: 180; year: 1968; month: 6; day: 28; verbatimEventDate: 28-Jun-68; **Record Level:** modified: 2015-01-08T00:00:00.000+0000; rights: http://creativecommons.org/licenses/by-nc/3.0/; rightsHolder: Museum of Texas Tech University; accessRights: CC BY-NC (Attribution-Non-Commercial); collectionID: d4e788b4-5d52-47a3-873e-227c6df49c7b; institutionCode: TTU; collectionCode: TTU-Z; basisOfRecord: PRESERVED_SPECIMEN
- **Type status:** Other material. **Occurrence:** occurrenceDetails: http://api.gbif.org/v1/occurrence/search?occurrenceId=ecf5848d-2f28-437d-9c9a-314aa8859014; catalogNumber: TTU-Z_219021; recordedBy: Robert Elkerson; individualCount: 1; preparations: pinned; otherCatalogNumbers: TTU1997-058; occurrenceID: ecf5848d-2f28-437d-9c9a-314aa8859014; **Taxon:** taxonID: 8801; scientificName: Compsusauricephalus Say, 1824; kingdom: Animalia; phylum: Arthropoda; class: Insecta; order: Coleoptera; family: Curculionidae; genus: Compsus; specificEpithet: auricephalus; taxonRank: SPECIES; taxonomicStatus: ACCEPTED; **Location:** country: United States of America; countryCode: US; stateProvince: Texas; county: Motley; decimalLatitude: 34.074095; decimalLongitude: -100.779829; geodeticDatum: WGS84; coordinateUncertaintyInMeters: 43365; georeferenceRemarks: 89 High MOTLEY COUNTY; **Event:** eventDate: 1968-06-25T00:00:00; startDayOfYear: 177; year: 1968; month: 6; day: 25; verbatimEventDate: 25-Jun-68; **Record Level:** modified: 2015-01-08T00:00:00.000+0000; rights: http://creativecommons.org/licenses/by-nc/3.0/; rightsHolder: Museum of Texas Tech University; accessRights: CC BY-NC (Attribution-Non-Commercial); collectionID: d4e788b4-5d52-47a3-873e-227c6df49c7b; institutionCode: TTU; collectionCode: TTU-Z; basisOfRecord: PRESERVED_SPECIMEN
- **Type status:** Other material. **Occurrence:** occurrenceDetails: http://api.gbif.org/v1/occurrence/search?occurrenceId=ea7be5d1-0592-4d4e-965b-9c7cf8674ed4; catalogNumber: TTU-Z_219022; recordedBy: C. R. Ward; individualCount: 1; preparations: pinned; otherCatalogNumbers: TTU1997-058; occurrenceID: ea7be5d1-0592-4d4e-965b-9c7cf8674ed4; **Taxon:** taxonID: 8801; scientificName: Compsusauricephalus Say, 1824; kingdom: Animalia; phylum: Arthropoda; class: Insecta; order: Coleoptera; family: Curculionidae; genus: Compsus; specificEpithet: auricephalus; taxonRank: SPECIES; taxonomicStatus: ACCEPTED; **Location:** country: United States of America; countryCode: US; stateProvince: Texas; county: Real; locality: Camp Wood; decimalLatitude: 29.66917; decimalLongitude: -100.01194; geodeticDatum: WGS84; coordinateUncertaintyInMeters: 952; georeferenceSources: GeoLocate; **Event:** eventDate: 1968-06-01T00:00:00; startDayOfYear: 153; year: 1968; month: 6; day: 1; verbatimEventDate: 1 VI 1968; **Record Level:** modified: 2015-01-08T00:00:00.000+0000; rights: http://creativecommons.org/licenses/by-nc/3.0/; rightsHolder: Museum of Texas Tech University; accessRights: CC BY-NC (Attribution-Non-Commercial); collectionID: d4e788b4-5d52-47a3-873e-227c6df49c7b; institutionCode: TTU; collectionCode: TTU-Z; basisOfRecord: PRESERVED_SPECIMEN
- **Type status:** Other material. **Occurrence:** occurrenceDetails: http://api.gbif.org/v1/occurrence/search?occurrenceId=9a3e6e5a-4517-4a56-8b7f-3b98a0e6d3b9; catalogNumber: TTU-Z_219301; recordedBy: R. R. Rogers; individualCount: 1; preparations: pinned; otherCatalogNumbers: TTU1997-058; occurrenceID: 9a3e6e5a-4517-4a56-8b7f-3b98a0e6d3b9; **Taxon:** taxonID: 8801; scientificName: Compsusauricephalus Say, 1824; kingdom: Animalia; phylum: Arthropoda; class: Insecta; order: Coleoptera; family: Curculionidae; genus: Compsus; specificEpithet: auricephalus; taxonRank: SPECIES; taxonomicStatus: ACCEPTED; **Location:** country: United States of America; countryCode: US; stateProvince: Texas; county: Jim Wells; locality: Alice; decimalLatitude: 27.75194; decimalLongitude: -98.06944; geodeticDatum: WGS84; coordinateUncertaintyInMeters: 6335; georeferenceSources: GeoLocate; **Event:** eventDate: 1968-04-08T00:00:00; startDayOfYear: 99; year: 1968; month: 4; day: 8; verbatimEventDate: 4/8/1968; **Record Level:** modified: 2015-01-08T00:00:00.000+0000; rights: http://creativecommons.org/licenses/by-nc/3.0/; rightsHolder: Museum of Texas Tech University; accessRights: CC BY-NC (Attribution-Non-Commercial); collectionID: d4e788b4-5d52-47a3-873e-227c6df49c7b; institutionCode: TTU; collectionCode: TTU-Z; basisOfRecord: PRESERVED_SPECIMEN
- **Type status:** Other material. **Occurrence:** occurrenceDetails: http://api.gbif.org/v1/occurrence/search?occurrenceId=f20cea5b-2318-465b-aaa0-947e63f12e64; catalogNumber: TTU-Z_219308; recordedBy: J. A. Campbell; individualCount: 1; preparations: pinned; otherCatalogNumbers: TTU1997-058; occurrenceID: f20cea5b-2318-465b-aaa0-947e63f12e64; **Taxon:** taxonID: 8801; scientificName: Compsusauricephalus Say, 1824; kingdom: Animalia; phylum: Arthropoda; class: Insecta; order: Coleoptera; family: Curculionidae; genus: Compsus; specificEpithet: auricephalus; taxonRank: SPECIES; taxonomicStatus: ACCEPTED; **Location:** country: United States of America; countryCode: US; stateProvince: Texas; county: Mason; locality: 1 mi. south of Mason; decimalLatitude: 30.734094; decimalLongitude: -99.23028; geodeticDatum: WGS84; coordinateUncertaintyInMeters: 5405; georeferenceSources: GeoLocate; **Event:** eventDate: 1970-10-01T00:00:00; startDayOfYear: 274; endDayOfYear: -30; year: 1970; month: 10; day: 1; verbatimEventDate: X - 1 - 70; **Record Level:** modified: 2015-01-08T00:00:00.000+0000; rights: http://creativecommons.org/licenses/by-nc/3.0/; rightsHolder: Museum of Texas Tech University; accessRights: CC BY-NC (Attribution-Non-Commercial); collectionID: d4e788b4-5d52-47a3-873e-227c6df49c7b; institutionCode: TTU; collectionCode: TTU-Z; basisOfRecord: PRESERVED_SPECIMEN
- **Type status:** Other material. **Occurrence:** occurrenceDetails: http://api.gbif.org/v1/occurrence/search?occurrenceId=483419bc-e5e1-4478-aace-5a5394fdd918; catalogNumber: TTU-Z_219310; occurrenceRemarks: mesquite Project Texas Tech University; recordNumber: 219310; recordedBy: L. B. O'Brien; individualCount: 1; lifeStage: ADULT; preparations: pinned; otherCatalogNumbers: TTU1997-058; occurrenceID: 483419bc-e5e1-4478-aace-5a5394fdd918; **Taxon:** taxonID: 8801; scientificName: Compsusauricephalus Say, 1824; kingdom: Animalia; phylum: Arthropoda; class: Insecta; order: Coleoptera; family: Curculionidae; genus: Compsus; specificEpithet: auricephalus; taxonRank: SPECIES; taxonomicStatus: ACCEPTED; **Location:** country: United States of America; countryCode: US; stateProvince: Texas; county: San Patricio; locality: Welder Wildlife Refuge; verbatimElevation: 800'; decimalLatitude: 28.122069; decimalLongitude: -97.442532; geodeticDatum: WGS84; coordinateUncertaintyInMeters: 100; georeferencedBy: alexa.davis (2014-07-31 14:47:25); georeferenceSources: georef batch tool 2014-07-31; GeoLocate; georeferenceVerificationStatus: reviewed - high confidence; **Identification:** identifiedBy: C. W. O'Brien; dateIdentified: 1972-01-01T00:00:00; **Event:** eventDate: 1971-04-02T00:00:00; startDayOfYear: 92; year: 1971; month: 4; day: 2; verbatimEventDate: IV-2-1971; **Record Level:** modified: 2014-07-31T00:00:00.000+0000; rights: http://creativecommons.org/licenses/by-nc/3.0/; rightsHolder: Museum of Texas Tech University; accessRights: CC BY-NC (Attribution-Non-Commercial); collectionID: d4e788b4-5d52-47a3-873e-227c6df49c7b; institutionCode: TTU; collectionCode: TTU-Z; basisOfRecord: PRESERVED_SPECIMEN
- **Type status:** Other material. **Occurrence:** occurrenceDetails: http://api.gbif.org/v1/occurrence/search?occurrenceId=1314bdaa-cf31-47ce-9102-9f4659b8c7d4; catalogNumber: TTU-Z_219023; recordedBy: K. Kattner; individualCount: 1; preparations: pinned; otherCatalogNumbers: TTU1997-058; occurrenceID: 1314bdaa-cf31-47ce-9102-9f4659b8c7d4; **Taxon:** taxonID: 8801; scientificName: Compsusauricephalus Say, 1824; kingdom: Animalia; phylum: Arthropoda; class: Insecta; order: Coleoptera; family: Curculionidae; genus: Compsus; specificEpithet: auricephalus; taxonRank: SPECIES; taxonomicStatus: ACCEPTED; **Location:** country: United States of America; countryCode: US; stateProvince: Texas; county: Jim Wells; decimalLatitude: 27.731338; decimalLongitude: -98.089886; geodeticDatum: WGS84; coordinateUncertaintyInMeters: 57990; georeferenceRemarks: 92 High JIM WELLS COUNTY; **Event:** eventDate: 1969-04-03T00:00:00; startDayOfYear: 93; year: 1969; month: 4; day: 3; verbatimEventDate: 3-Apr-69; **Record Level:** modified: 2015-01-08T00:00:00.000+0000; rights: http://creativecommons.org/licenses/by-nc/3.0/; rightsHolder: Museum of Texas Tech University; accessRights: CC BY-NC (Attribution-Non-Commercial); collectionID: d4e788b4-5d52-47a3-873e-227c6df49c7b; institutionCode: TTU; collectionCode: TTU-Z; basisOfRecord: PRESERVED_SPECIMEN
- **Type status:** Other material. **Occurrence:** occurrenceDetails: http://api.gbif.org/v1/occurrence/search?occurrenceId=5501285b-d654-4b55-b188-6155bd373f85; catalogNumber: TTU-Z_050014; occurrenceRemarks: On Mesquite Tree; recordedBy: James C. Cokendolpher; J.Creel; individualCount: 1; lifeStage: ADULT; preparations: dry pinned; otherCatalogNumbers: TTU1997-058; occurrenceID: 5501285b-d654-4b55-b188-6155bd373f85; **Taxon:** taxonID: 8801; scientificName: Compsusauricephalus Say, 1824; kingdom: Animalia; phylum: Arthropoda; class: Insecta; order: Coleoptera; family: Curculionidae; genus: Compsus; specificEpithet: auricephalus; taxonRank: SPECIES; taxonomicStatus: ACCEPTED; **Location:** country: United States of America; countryCode: US; stateProvince: Texas; county: Garza; locality: Road side Park on Caprock just NE of Post; decimalLatitude: 33.19083; decimalLongitude: -101.37778; geodeticDatum: WGS84; coordinateUncertaintyInMeters: 2337; georeferencedBy: Alex Gregg (2014-07-30 12:00:49); georeferenceSources: georef batch tool 2014-07-30; GeoLocate; georeferenceVerificationStatus: reviewed - high confidence; **Identification:** identifiedBy: R. S. Anderson; dateIdentified: 2017-01-01T00:00:00; **Event:** eventDate: 2007-05-16T00:00:00; startDayOfYear: 136; year: 2007; month: 5; day: 16; verbatimEventDate: 16-V-2007; **Record Level:** modified: 2015-01-08T00:00:00.000+0000; rights: http://creativecommons.org/licenses/by-nc/3.0/; rightsHolder: Museum of Texas Tech University; accessRights: CC BY-NC (Attribution-Non-Commercial); collectionID: d4e788b4-5d52-47a3-873e-227c6df49c7b; institutionCode: TTU; collectionCode: TTU-Z; basisOfRecord: PRESERVED_SPECIMEN
- **Type status:** Other material. **Occurrence:** occurrenceDetails: http://api.gbif.org/v1/occurrence/search?occurrenceId=bed89b36-3a63-405b-879e-bf85666ded92; catalogNumber: TTU-Z_219288; recordedBy: L. B. Smith; individualCount: 1; lifeStage: ADULT; preparations: pinned; otherCatalogNumbers: TTU1997-058; occurrenceID: bed89b36-3a63-405b-879e-bf85666ded92; **Taxon:** taxonID: 8801; scientificName: Compsusauricephalus Say, 1824; kingdom: Animalia; phylum: Arthropoda; class: Insecta; order: Coleoptera; family: Curculionidae; genus: Compsus; specificEpithet: auricephalus; taxonRank: SPECIES; taxonomicStatus: ACCEPTED; **Location:** country: United States of America; countryCode: US; stateProvince: Texas; county: Dickens; locality: 7-Bar Ranch; decimalLatitude: 33.758961; decimalLongitude: -100.755789; geodeticDatum: WGS84; coordinateUncertaintyInMeters: 100; georeferencedBy: luis.tirado (2014-08-01 20:13:56); georeferenceSources: georef batch tool 2014-08-01; GeoLocate; georeferenceVerificationStatus: reviewed - high confidence; **Identification:** identifiedBy: L. B. Smith; **Event:** eventDate: 1972-05-17T00:00:00; startDayOfYear: 138; year: 1972; month: 5; day: 17; verbatimEventDate: 5/17/1972; **Record Level:** modified: 2015-01-08T00:00:00.000+0000; rights: http://creativecommons.org/licenses/by-nc/3.0/; rightsHolder: Museum of Texas Tech University; accessRights: CC BY-NC (Attribution-Non-Commercial); collectionID: d4e788b4-5d52-47a3-873e-227c6df49c7b; institutionCode: TTU; collectionCode: TTU-Z; basisOfRecord: PRESERVED_SPECIMEN
- **Type status:** Other material. **Occurrence:** occurrenceDetails: http://api.gbif.org/v1/occurrence/search?occurrenceId=78a280bd-fead-404d-afba-9ab04e1ff1ce; catalogNumber: TTU-Z_219287; recordedBy: L. B. Smith; individualCount: 1; lifeStage: ADULT; preparations: pinned; otherCatalogNumbers: TTU1997-058; occurrenceID: 78a280bd-fead-404d-afba-9ab04e1ff1ce; **Taxon:** taxonID: 8801; scientificName: Compsusauricephalus Say, 1824; kingdom: Animalia; phylum: Arthropoda; class: Insecta; order: Coleoptera; family: Curculionidae; genus: Compsus; specificEpithet: auricephalus; taxonRank: SPECIES; taxonomicStatus: ACCEPTED; **Location:** country: United States of America; countryCode: US; stateProvince: Texas; county: Dickens; locality: 7-Bar Ranch; decimalLatitude: 33.758961; decimalLongitude: -100.755789; geodeticDatum: WGS84; coordinateUncertaintyInMeters: 100; georeferencedBy: luis.tirado (2014-08-01 20:13:56); georeferenceSources: georef batch tool 2014-08-01; GeoLocate; georeferenceVerificationStatus: reviewed - high confidence; **Identification:** identifiedBy: L. B. Smith; **Event:** eventDate: 1972-05-17T00:00:00; startDayOfYear: 138; year: 1972; month: 5; day: 17; verbatimEventDate: 5/17/1972; **Record Level:** modified: 2015-01-08T00:00:00.000+0000; rights: http://creativecommons.org/licenses/by-nc/3.0/; rightsHolder: Museum of Texas Tech University; accessRights: CC BY-NC (Attribution-Non-Commercial); collectionID: d4e788b4-5d52-47a3-873e-227c6df49c7b; institutionCode: TTU; collectionCode: TTU-Z; basisOfRecord: PRESERVED_SPECIMEN
- **Type status:** Other material. **Occurrence:** occurrenceDetails: http://api.gbif.org/v1/occurrence/search?occurrenceId=8bd7da64-4d84-4260-b606-34cef413c301; catalogNumber: TTU-Z_219295; recordedBy: Bryant Mather; individualCount: 1; preparations: pinned; otherCatalogNumbers: TTU1997-058; occurrenceID: 8bd7da64-4d84-4260-b606-34cef413c301; **Taxon:** taxonID: 8801; scientificName: Compsusauricephalus Say, 1824; kingdom: Animalia; phylum: Arthropoda; class: Insecta; order: Coleoptera; family: Curculionidae; genus: Compsus; specificEpithet: auricephalus; taxonRank: SPECIES; taxonomicStatus: ACCEPTED; **Location:** country: United States of America; countryCode: US; stateProvince: Mississippi; county: Warren; decimalLatitude: 32.35723; decimalLongitude: -90.852011; geodeticDatum: WGS84; coordinateUncertaintyInMeters: 43674; georeferenceRemarks: 89 High WARREN COUNTY; **Identification:** identifiedBy: C.W. O'Brien; dateIdentified: 1974-01-01T00:00:00; **Event:** eventDate: 1973-06-01T00:00:00; startDayOfYear: 152; year: 1973; month: 6; day: 1; verbatimEventDate: 1-Jun-73; **Record Level:** modified: 2015-01-08T00:00:00.000+0000; rights: http://creativecommons.org/licenses/by-nc/3.0/; rightsHolder: Museum of Texas Tech University; accessRights: CC BY-NC (Attribution-Non-Commercial); collectionID: d4e788b4-5d52-47a3-873e-227c6df49c7b; institutionCode: TTU; collectionCode: TTU-Z; basisOfRecord: PRESERVED_SPECIMEN
- **Type status:** Other material. **Occurrence:** occurrenceDetails: http://api.gbif.org/v1/occurrence/search?occurrenceId=6d0aa9ae-9f7e-4226-952d-47ebf9bfdc71; catalogNumber: TTU-Z_219296; recordedBy: Bryant Mather; individualCount: 1; preparations: pinned; otherCatalogNumbers: TTU1997-058; occurrenceID: 6d0aa9ae-9f7e-4226-952d-47ebf9bfdc71; **Taxon:** taxonID: 8801; scientificName: Compsusauricephalus Say, 1824; kingdom: Animalia; phylum: Arthropoda; class: Insecta; order: Coleoptera; family: Curculionidae; genus: Compsus; specificEpithet: auricephalus; taxonRank: SPECIES; taxonomicStatus: ACCEPTED; **Location:** country: United States of America; countryCode: US; stateProvince: Mississippi; county: Warren; decimalLatitude: 32.35723; decimalLongitude: -90.852011; geodeticDatum: WGS84; coordinateUncertaintyInMeters: 43674; georeferenceRemarks: 89 High WARREN COUNTY; **Identification:** identifiedBy: C.W. O'Brien; dateIdentified: 1974-01-01T00:00:00; **Event:** eventDate: 1973-06-18T00:00:00; startDayOfYear: 169; year: 1973; month: 6; day: 18; verbatimEventDate: 18-Jun-73; **Record Level:** modified: 2015-01-08T00:00:00.000+0000; rights: http://creativecommons.org/licenses/by-nc/3.0/; rightsHolder: Museum of Texas Tech University; accessRights: CC BY-NC (Attribution-Non-Commercial); collectionID: d4e788b4-5d52-47a3-873e-227c6df49c7b; institutionCode: TTU; collectionCode: TTU-Z; basisOfRecord: PRESERVED_SPECIMEN
- **Type status:** Other material. **Occurrence:** occurrenceDetails: http://api.gbif.org/v1/occurrence/search?occurrenceId=b679d423-1867-4788-ac0f-45c56fbf0ded; catalogNumber: TTU-Z_219289; recordedBy: R. Morris II.; individualCount: 1; preparations: pinned; otherCatalogNumbers: TTU1997-058; occurrenceID: b679d423-1867-4788-ac0f-45c56fbf0ded; **Taxon:** taxonID: 8801; scientificName: Compsusauricephalus Say, 1824; kingdom: Animalia; phylum: Arthropoda; class: Insecta; order: Coleoptera; family: Curculionidae; genus: Compsus; specificEpithet: auricephalus; taxonRank: SPECIES; taxonomicStatus: ACCEPTED; **Location:** country: United States of America; countryCode: US; stateProvince: Texas; county: Cameron; locality: Palmetto Hill, 10 m w Boca Chica; decimalLatitude: 25.977545; decimalLongitude: -97.351891; geodeticDatum: WGS84; coordinateUncertaintyInMeters: 500; georeferencedBy: luis.tirado (2014-08-01 20:01:53); georeferenceSources: georef batch tool 2014-08-01; GeoLocate; georeferenceVerificationStatus: reviewed - high confidence; **Identification:** identifiedBy: Downie; **Event:** eventDate: 1985-10-13T00:00:00; startDayOfYear: 286; year: 1985; month: 10; day: 13; verbatimEventDate: Oct. 13 1985; **Record Level:** modified: 2015-01-08T00:00:00.000+0000; rights: http://creativecommons.org/licenses/by-nc/3.0/; rightsHolder: Museum of Texas Tech University; accessRights: CC BY-NC (Attribution-Non-Commercial); collectionID: d4e788b4-5d52-47a3-873e-227c6df49c7b; institutionCode: TTU; collectionCode: TTU-Z; basisOfRecord: PRESERVED_SPECIMEN
- **Type status:** Other material. **Occurrence:** occurrenceDetails: http://api.gbif.org/v1/occurrence/search?occurrenceId=8e647254-b380-4d0c-957f-b0ac420bd757; catalogNumber: TTU-Z_219290; recordedBy: R. Morris II.; individualCount: 1; preparations: pinned; otherCatalogNumbers: TTU1997-058; occurrenceID: 8e647254-b380-4d0c-957f-b0ac420bd757; **Taxon:** taxonID: 8801; scientificName: Compsusauricephalus Say, 1824; kingdom: Animalia; phylum: Arthropoda; class: Insecta; order: Coleoptera; family: Curculionidae; genus: Compsus; specificEpithet: auricephalus; taxonRank: SPECIES; taxonomicStatus: ACCEPTED; **Location:** country: United States of America; countryCode: US; stateProvince: Texas; county: Cameron; locality: Palmetto Hill, 10 m w Boca Chica; decimalLatitude: 25.977545; decimalLongitude: -97.351891; geodeticDatum: WGS84; coordinateUncertaintyInMeters: 500; georeferencedBy: luis.tirado (2014-08-01 20:01:53); georeferenceSources: georef batch tool 2014-08-01; GeoLocate; georeferenceVerificationStatus: reviewed - high confidence; **Identification:** identifiedBy: C.W. O'Brien; dateIdentified: 1974-01-01T00:00:00; **Event:** eventDate: 1985-10-13T00:00:00; startDayOfYear: 286; year: 1985; month: 10; day: 13; verbatimEventDate: Oct. 13 1985; **Record Level:** modified: 2015-01-08T00:00:00.000+0000; rights: http://creativecommons.org/licenses/by-nc/3.0/; rightsHolder: Museum of Texas Tech University; accessRights: CC BY-NC (Attribution-Non-Commercial); collectionID: d4e788b4-5d52-47a3-873e-227c6df49c7b; institutionCode: TTU; collectionCode: TTU-Z; basisOfRecord: PRESERVED_SPECIMEN
- **Type status:** Other material. **Occurrence:** occurrenceDetails: http://api.gbif.org/v1/occurrence/search?occurrenceId=b15e19a4-a01e-4566-9c18-bbe083e0ecc0; catalogNumber: TTU-Z_219291; recordedBy: R. Morris II.; individualCount: 1; preparations: pinned; otherCatalogNumbers: TTU1997-058; occurrenceID: b15e19a4-a01e-4566-9c18-bbe083e0ecc0; **Taxon:** taxonID: 8801; scientificName: Compsusauricephalus Say, 1824; kingdom: Animalia; phylum: Arthropoda; class: Insecta; order: Coleoptera; family: Curculionidae; genus: Compsus; specificEpithet: auricephalus; taxonRank: SPECIES; taxonomicStatus: ACCEPTED; **Location:** country: United States of America; countryCode: US; stateProvince: Texas; county: Cameron; locality: Palmetto Hill, 10 m w Boca Chica; decimalLatitude: 25.977545; decimalLongitude: -97.351891; geodeticDatum: WGS84; coordinateUncertaintyInMeters: 500; georeferencedBy: luis.tirado (2014-08-01 20:01:53); georeferenceSources: georef batch tool 2014-08-01; GeoLocate; georeferenceVerificationStatus: reviewed - high confidence; **Identification:** identifiedBy: C.W. O'Brien; dateIdentified: 1974-01-01T00:00:00; **Event:** eventDate: 1985-10-13T00:00:00; startDayOfYear: 286; year: 1985; month: 10; day: 13; verbatimEventDate: Oct. 13 1985; **Record Level:** modified: 2015-01-08T00:00:00.000+0000; rights: http://creativecommons.org/licenses/by-nc/3.0/; rightsHolder: Museum of Texas Tech University; accessRights: CC BY-NC (Attribution-Non-Commercial); collectionID: d4e788b4-5d52-47a3-873e-227c6df49c7b; institutionCode: TTU; collectionCode: TTU-Z; basisOfRecord: PRESERVED_SPECIMEN
- **Type status:** Other material. **Occurrence:** occurrenceDetails: http://api.gbif.org/v1/occurrence/search?occurrenceId=b534485d-87b2-45d8-8fd7-ef96f9d0e34d; catalogNumber: TTU-Z_219292; recordedBy: R. Morris II.; individualCount: 1; preparations: pinned; otherCatalogNumbers: TTU1997-058; occurrenceID: b534485d-87b2-45d8-8fd7-ef96f9d0e34d; **Taxon:** taxonID: 8801; scientificName: Compsusauricephalus Say, 1824; kingdom: Animalia; phylum: Arthropoda; class: Insecta; order: Coleoptera; family: Curculionidae; genus: Compsus; specificEpithet: auricephalus; taxonRank: SPECIES; taxonomicStatus: ACCEPTED; **Location:** country: United States of America; countryCode: US; stateProvince: Texas; county: Cameron; locality: Palmetto Hill, 10 m w Boca Chica; decimalLatitude: 25.977545; decimalLongitude: -97.351891; geodeticDatum: WGS84; coordinateUncertaintyInMeters: 500; georeferencedBy: luis.tirado (2014-08-01 20:01:53); georeferenceSources: georef batch tool 2014-08-01; GeoLocate; georeferenceVerificationStatus: reviewed - high confidence; **Identification:** identifiedBy: C.W. O'Brien; dateIdentified: 1974-01-01T00:00:00; **Event:** eventDate: 1985-10-13T00:00:00; startDayOfYear: 286; year: 1985; month: 10; day: 13; verbatimEventDate: Oct. 13 1985; **Record Level:** modified: 2015-01-08T00:00:00.000+0000; rights: http://creativecommons.org/licenses/by-nc/3.0/; rightsHolder: Museum of Texas Tech University; accessRights: CC BY-NC (Attribution-Non-Commercial); collectionID: d4e788b4-5d52-47a3-873e-227c6df49c7b; institutionCode: TTU; collectionCode: TTU-Z; basisOfRecord: PRESERVED_SPECIMEN
- **Type status:** Other material. **Occurrence:** occurrenceDetails: http://api.gbif.org/v1/occurrence/search?occurrenceId=92e14681-3a2f-4a7d-80a2-1bade6908523; catalogNumber: TTU-Z_219293; recordedBy: R. Morris II.; individualCount: 1; preparations: pinned; otherCatalogNumbers: TTU1997-058; occurrenceID: 92e14681-3a2f-4a7d-80a2-1bade6908523; **Taxon:** taxonID: 8801; scientificName: Compsusauricephalus Say, 1824; kingdom: Animalia; phylum: Arthropoda; class: Insecta; order: Coleoptera; family: Curculionidae; genus: Compsus; specificEpithet: auricephalus; taxonRank: SPECIES; taxonomicStatus: ACCEPTED; **Location:** country: United States of America; countryCode: US; stateProvince: Texas; county: Cameron; locality: Palmetto Hill, 10 m w Boca Chica; decimalLatitude: 25.977545; decimalLongitude: -97.351891; geodeticDatum: WGS84; coordinateUncertaintyInMeters: 500; georeferencedBy: luis.tirado (2014-08-01 20:01:53); georeferenceSources: georef batch tool 2014-08-01; GeoLocate; georeferenceVerificationStatus: reviewed - high confidence; **Identification:** identifiedBy: C.W. O'Brien; dateIdentified: 1974-01-01T00:00:00; **Event:** eventDate: 1985-10-13T00:00:00; startDayOfYear: 286; year: 1985; month: 10; day: 13; verbatimEventDate: Oct. 13 1985; **Record Level:** modified: 2015-01-08T00:00:00.000+0000; rights: http://creativecommons.org/licenses/by-nc/3.0/; rightsHolder: Museum of Texas Tech University; accessRights: CC BY-NC (Attribution-Non-Commercial); collectionID: d4e788b4-5d52-47a3-873e-227c6df49c7b; institutionCode: TTU; collectionCode: TTU-Z; basisOfRecord: PRESERVED_SPECIMEN
- **Type status:** Other material. **Occurrence:** occurrenceDetails: http://api.gbif.org/v1/occurrence/search?occurrenceId=0a6c95e9-5f56-4c10-9cd7-a116973b7bb5; catalogNumber: TTU-Z_219294; recordedBy: R. Morris II.; individualCount: 1; preparations: pinned; otherCatalogNumbers: TTU1997-058; occurrenceID: 0a6c95e9-5f56-4c10-9cd7-a116973b7bb5; **Taxon:** taxonID: 8801; scientificName: Compsusauricephalus Say, 1824; kingdom: Animalia; phylum: Arthropoda; class: Insecta; order: Coleoptera; family: Curculionidae; genus: Compsus; specificEpithet: auricephalus; taxonRank: SPECIES; taxonomicStatus: ACCEPTED; **Location:** country: United States of America; countryCode: US; stateProvince: Texas; county: Cameron; locality: Palmetto Hill, 10 m w Boca Chica; decimalLatitude: 25.977545; decimalLongitude: -97.351891; geodeticDatum: WGS84; coordinateUncertaintyInMeters: 500; georeferencedBy: luis.tirado (2014-08-01 20:01:53); georeferenceSources: georef batch tool 2014-08-01; GeoLocate; georeferenceVerificationStatus: reviewed - high confidence; **Identification:** identifiedBy: C.W. O'Brien; dateIdentified: 1974-01-01T00:00:00; **Event:** eventDate: 1985-10-13T00:00:00; startDayOfYear: 286; year: 1985; month: 10; day: 13; verbatimEventDate: Oct. 13 1985; **Record Level:** modified: 2015-01-08T00:00:00.000+0000; rights: http://creativecommons.org/licenses/by-nc/3.0/; rightsHolder: Museum of Texas Tech University; accessRights: CC BY-NC (Attribution-Non-Commercial); collectionID: d4e788b4-5d52-47a3-873e-227c6df49c7b; institutionCode: TTU; collectionCode: TTU-Z; basisOfRecord: PRESERVED_SPECIMEN
- **Type status:** Other material. **Occurrence:** occurrenceDetails: http://api.gbif.org/v1/occurrence/search?occurrenceId=a46cfd85-0763-4003-9e75-e23cb53a20cc; catalogNumber: TTU-Z_224142; recordedBy: J. Hatchett; individualCount: 1; lifeStage: ADULT; preparations: dry pinned; otherCatalogNumbers: TTU1997-058; occurrenceID: a46cfd85-0763-4003-9e75-e23cb53a20cc; **Taxon:** taxonID: 8801; scientificName: Compsusauricephalus Say, 1824; kingdom: Animalia; phylum: Arthropoda; class: Insecta; order: Coleoptera; family: Curculionidae; genus: Compsus; specificEpithet: auricephalus; taxonRank: SPECIES; taxonomicStatus: ACCEPTED; **Location:** country: United States of America; countryCode: US; stateProvince: Texas; county: Lubbock; decimalLatitude: 33.610206; decimalLongitude: -101.820546; geodeticDatum: WGS84; coordinateUncertaintyInMeters: 41227; georeferenceRemarks: 90 High LUBBOCK COUNTY; **Event:** eventDate: 1951-04-11T00:00:00; startDayOfYear: 101; year: 1951; month: 4; day: 11; verbatimEventDate: 4/11/1951; **Record Level:** modified: 2015-01-08T00:00:00.000+0000; rights: http://creativecommons.org/licenses/by-nc/3.0/; rightsHolder: Museum of Texas Tech University; accessRights: CC BY-NC (Attribution-Non-Commercial); collectionID: d4e788b4-5d52-47a3-873e-227c6df49c7b; institutionCode: TTU; collectionCode: TTU-Z; basisOfRecord: PRESERVED_SPECIMEN
- **Type status:** Other material. **Occurrence:** occurrenceDetails: http://api.gbif.org/v1/occurrence/search?occurrenceId=d53c30d3-5f8f-4079-b90c-8c40b72a14e2; catalogNumber: TTU-Z_223955; individualCount: 1; lifeStage: ADULT; preparations: Pointed; otherCatalogNumbers: TTU1997-058; occurrenceID: d53c30d3-5f8f-4079-b90c-8c40b72a14e2; **Taxon:** taxonID: 8801; scientificName: Compsusauricephalus Say, 1824; kingdom: Animalia; phylum: Arthropoda; class: Insecta; order: Coleoptera; family: Curculionidae; genus: Compsus; specificEpithet: auricephalus; taxonRank: SPECIES; taxonomicStatus: ACCEPTED; **Location:** country: United States of America; countryCode: US; stateProvince: Texas; county: San Patricio; locality: Welder Wildlife Refuge, Pollita Lake; decimalLatitude: 28.11228; decimalLongitude: -97.41609; geodeticDatum: WGS84; coordinateUncertaintyInMeters: 500; georeferencedBy: Alex Gregg (2014-08-07 14:37:43); georeferenceSources: georef batch tool 2014-08-07; GeoLocate; georeferenceVerificationStatus: reviewed - high confidence; **Identification:** identifiedBy: J. Girón; dateIdentified: 2020-01-01T00:00:00; **Event:** samplingProtocol: at night; eventDate: 1971-04-03T00:00:00; startDayOfYear: 93; year: 1971; month: 4; day: 3; verbatimEventDate: IV-3-1971; **Record Level:** modified: 2015-01-08T00:00:00.000+0000; rights: http://creativecommons.org/licenses/by-nc/3.0/; rightsHolder: Museum of Texas Tech University; accessRights: CC BY-NC (Attribution-Non-Commercial); collectionID: d4e788b4-5d52-47a3-873e-227c6df49c7b; institutionCode: TTU; collectionCode: TTU-Z; basisOfRecord: PRESERVED_SPECIMEN
- **Type status:** Other material. **Occurrence:** occurrenceDetails: http://api.gbif.org/v1/occurrence/search?occurrenceId=c3ff87d1-25d3-4756-af25-3085e50fbe4b; catalogNumber: TTU-Z_224887; recordedBy: J. Boren; individualCount: 1; lifeStage: ADULT; preparations: Pointed; otherCatalogNumbers: TTU1997-058; occurrenceID: c3ff87d1-25d3-4756-af25-3085e50fbe4b; **Taxon:** taxonID: 8801; scientificName: Compsusauricephalus Say, 1824; kingdom: Animalia; phylum: Arthropoda; class: Insecta; order: Coleoptera; family: Curculionidae; genus: Compsus; specificEpithet: auricephalus; taxonRank: SPECIES; taxonomicStatus: ACCEPTED; **Location:** country: United States of America; countryCode: US; stateProvince: Texas; county: Garza; decimalLatitude: 33.179876; decimalLongitude: -101.298424; geodeticDatum: WGS84; coordinateUncertaintyInMeters: 41220; georeferenceRemarks: 88 High GARZA COUNTY; **Event:** eventDate: 1956-04-21T00:00:00; startDayOfYear: 112; year: 1956; month: 4; day: 21; verbatimEventDate: 4/21/1956; **Record Level:** modified: 2015-01-08T00:00:00.000+0000; rights: http://creativecommons.org/licenses/by-nc/3.0/; rightsHolder: Museum of Texas Tech University; accessRights: CC BY-NC (Attribution-Non-Commercial); collectionID: d4e788b4-5d52-47a3-873e-227c6df49c7b; institutionCode: TTU; collectionCode: TTU-Z; basisOfRecord: PRESERVED_SPECIMEN

#### Description

Body length 8–12 mm, width 3–4 mm; shape oval, length/width ratio 2.4–2.6; greatest width near mid-length of elytra in males, near posterior third in females. Integument dark brown to black; coverage composed of densely and evenly arranged, overlapping scales (Figs 1, 3A–C), mostly to completely covering integument, subcircular to distally angulate, appressed; scales uniformly pearly white to light brown (Fig. 1C, Fig. 3A–C) or iridescent light green along body with iridescent pink along dorsal surface of head and along anterior and ventral surfaces of legs (Fig. 1A, B, Fig. 3F); seta-like narrow scales sparsely and evenly arranged, relatively short and thick, recurvate, pale white to translucent.

**Head.** Frons nearly flat, only very slightly transversally impressed at level of posterior margin of eyes; frons with deep, large median fovea (Fig. 4A, C, mf); surface of head densely covered by overlapping scales; scales apically angulate along base of head, progressing to oval anteriorly (Fig. 4B); curved, thick setae only along dorsal surface of head. Eyes in lateral view (Fig. 4B) tear-drop shaped, 1.5-times longer than wide, with acute margin pointing antero-ventrally; eyes mostly lateral, separated from anterolateral margin of prothorax by distance slightly shorter than greatest width of eye; in dorsal view (Fig. 4A, E–G), eyes moderately convex, with inner margins slightly oblique, interocular distance 4.6-times maximum width of eye.

**Rostrum.** Only very slightly wider than long (Fig. 4A, E–G), anteriorly slightly widened apicad of antennal insertion; shape in cross-section subrectangular. In dorsal view (Fig. 4A, E–G), outline of rostrum subquadrate; dorsolateral margins (Fig. 4A, dlm; dorsal margin of scrobe in dorsal view) arcuate, anteriorly and posteriorly diverging, elevated (see Fig. 4C); apical margin deeply emarginate, somewhat sinuate (Fig. 4C, apm). Nasal plate (Fig. 4C, np; see Vaurie 1963, Girón and Howden 2019) moderate in size, with depressed surface, positioned nearly perpendicular to surface of epistoma, with posterior margin bluntly elevated. Epistoma (Fig. 4C, ep) distinctly depressed, with small, oval scales along posterolateral areas; posterior margin of epistoma transversely elevated. Epistomal setae (Fig. 4C, eps) moderately thick, long and dense, becoming longer apically, apicomedially pointing. Dorsal surface of rostrum with one deep, median sulcus (Fig. 4C, ms), anteriorly broadly widened forming large, elongate triangular depression, extending from posterior margin of epistoma to midpoint between eyes, reaching and somewhat fusing with median frontal fovea; with two short dorsolateral sulci (Fig. 4C, ds) positioned along posterior half of rostrum; ventrolateral margins (Fig. 4A, vlm) slightly arcuate, anteriorly diverging. Ventral margin of antennal scrobe, often fully visible in dorsal view of rostrum. Rostrum in lateral view (Fig. 4B) slightly and gradually expanded apically, with dorsal outline nearly straight along basal half; occipital suture (Fig. 4B, D, os) extending from anteroventral margin of eye to ventral mid-length of rostrum to meet anterior tentorial pit (Fig. 4D, atp), continuing anteriorly to near apex of rostrum; margins of mandibular incision with rather long, thick suberect setae (Fig. 4D); gular sutures (Fig. 4D, gs) basally fused together medially, separating at base of rostrum, extending to posterior tentorial pits (Fig. 4D, ptp). Antennal insertion near anterior third of rostrum. Scrobe lateral (Fig. 4B), slightly arcuate, gradually and slightly expanded and shallower towards eye, initiating in apicodorsal region, terminating in basimedial region of rostrum, with dorsal margin well-defined throughout, ending at mid-length of anterior margin of eye; ventral margin of scrobe ending near mid-length of rostrum; scrobe covered with scales along posterodorsal areas (Fig. 4B).

**Mouthparts.** Mandibles with multiple setae along dorsal, outer and ventral areas surrounding scar (Fig. 4B–D; few scales on outer surface; mandibular scar protruded and sharply raised (Fig. 4C; see also Girón and Franz 2012, character 14); deciduous processes (Fig. 4E, G, dp) large, sickle-shaped, nearly symmetrical, nearly as long as greatest dorsal width of rostrum. Maxillae (Fig. 5A) with cardo (cd) longitudinally twisted; lateral margin of stipes (st) with five or six setae of various lengths along basal half of margin and two along distal region, ventral surface with three thick setae near mesal margin; galeo-lacinial complex (Fig. 5A, ga+la; not considering thick apical setae) extending to apex of maxillary palpomere 2 (Fig. 5A, mpm2), apical region round, mesal margin with basal tuft of very long setae (nearly as long as longest length of cardo) and one lacinial tooth (Fig. 5A, lat), apical region with four to five thick and flattened major galeal teeth (Fig. 5A, gat), accompanied by numerous minor teeth and dense, thick setae of various lengths; palpiger (Fig. 5A, pg) membranous along apical half, basal region more sclerotised, with transverse row of setae along margin of scerotised region of dorsal face; maxillary palps with three palpomeres; maxillary palpomere 1 (Fig. 5A, mpm1) nearly 1.5-times longer than 3 (Fig. 5A, mpm3), 2-times longer than 2 (Fig. 5A, mpm2), with oblique apical margin; apicolateral margin of palpomere 1 with one ventral seta; apical margin of palpomere 2 with one dorsal and one ventral seta; palpomere 3 slightly longer than wide, with several longitudinal sulci and a crown of papillae across apical surface. Labium with prementum (Fig. 4D, prm) entirely covering maxillary palps; sub-rectangular, surface reticulate, with apical margin medially slightly angulate; ventral surface basi-medially depressed with long setae along each side; ligula narrow (Fig. 5C, lg), with one strong long mesal seta at apex; labial palps (Fig. 5B, C) with three palpomeres; labial palpomeres 1 and 3 similar in length (Fig. 5B, C lpm1, lpm3, respectively), palpomere 2 (Fig. 5B, C lpm2) only slightly shorter; apico-ventral margins of palpomeres 1 and 2 each with one seta, palpomere 3 (Fig. 5B, C lpm3) slightly longer than wide, with basiconical sensillae across apical surface.

**Antennae.** With 12 antennomeres (Fig. 4A, B). Scape slightly bent along mid section, extending beyond posterior margin of eye, not reaching anterolateral margin of prothorax, passing over ventral fifth of eye (in resting position), densely covered with scales, with sparse, thick, white setae. Funicle with seven antennomeres, 1.3-times longer than scape; funicular antennomeres clavate, progressing from distinctly to slightly elongate; funicular antennomere 2 slightly longer than 1; 1 slightly longer than 3; 4 and 5 similar in shape, shorter than 3; 6, 7 and 8 similar in shape, shorter than 5. Club with four distinct visible antennomeres (terminal annulation fully differentiated, see Fig. 4B), nearly as long as funicular antennomeres 5–7 combined, nearly 3-times longer than wide, densely covered by translucent setae; club antennomeres gradually decreasing in length towards apex.

**Thorax.** Pronotum somewhat trapezoid (Fig. 3A), 1.3-times wider at base than at apex, 1.2-times wider at base than long; pronotum nearly 0.3-times length of elytra, with greatest width at base; dorsolateral margins anteriorly converging, posteriorly subparallel; posterior margin only very slightly bisinuate, with lateral areas depressed, and covered by densely arranged plumose setae. Dorsal surface of pronotum medially flattened to depressed, with dorsolateral, longitudinal, broad elevations, irregularly foveate to punctate; lateral surfaces flattened, irregularly foveate to punctate; scales appressed, evenly and densely distributed for the most part; with scattered recurvate setae, anteriorly or medially directed; median area of pronotum often posteriorly depressed. Prothorax in lateral view (Fig. 3B) subquadrate to trapezoid; anterior margin straight and slightly oblique, without postocular lobe, fringed with angulate scales and sometimes with plumose setae projecting anteriorly; postocular vibrissae absent; dorsolateral surface broadly and longitudinally elevated posteriad coxal insertion; posterolateral surface depressed. Metascutum partially covered with plumose scales, especially along posterior margin. Scutellar shield subquadrate to elongate with round corners, covered by scales. Prosternum (Fig. 3C) slightly longer than mesosternite, similar in length to metasternite; prosternum with transverse anterior and posterior sulci; procoxal cavities contiguous, 0.7-times closer to anterior than to posterior margin of prosternum; anterior margin of prosternum fringed by regular setae, plumose setae and scales. Mesosternite (Fig. 3C) with intercoxal process slightly elevated, with recurvate setae; mesocoxal cavities separated by distance nearly 0.3-times greatest width of each mesocoxal cavity. Metasternite (Fig. 3C) with median sulcus short, shallow, Y-shaped, positioned near posterior margin; metacoxal cavities separated by distance nearly 0.8-times width of each mesocoxal cavity. Metendosternite (Fig. 5D) with stalk (sk) 1.5-times longer than each furcal arm (fa); stalk somewhat triangular (wider at junction with sternum, gradually narrowing towards crux (cr)); distal sheaths (ds) well developed, as wide as widest point of crux; anterior tendons (at) inserted at dorsal margin of distal sheaths, at mesal one-third between longitudinal flange (lf) and furcal arms; furcal arms somewhat triangular, diverging, with apex slightly explanate; hemiducti (hm) well developed, ventrolaterally pointing; posterior mesal sheath extending between dorsal margin of distal sheath and mid-length of stalk.

**Legs.** (Fig. 3B, C, F) Prothoracic legs slightly longer than mesothoracic legs, slightly shorter than metathoracic legs; with evenly spaced whitish, recurvate setae along femora, denser along tibiae, especially along antero-ventral margin. Profemur nearly 1.5-times longer than prothorax; profemur gradually enlarging to slightly apicad of midlength, with ventral margin basally straight, distally strongly bisinuate; unarmed, but surface ventrally slightly projected at profemoral expansion. Protibia slightly longer than profemur, slender, apically slightly expanded, with ventral margin slightly arcuate, especially along apical fourth; ventral margin without cuticular teeth, with row of translucent to whitish spiniform setae; protibial apex with anterior margin slightly arcuate, densely fringed by fine setae; mucro shorter than to similar in length to tarsal claw, surpassed by tuft of fine, long setae. Protarsomeres 1 and 3 similar in length, protarsomere 2 0.6-times length of protarsomere 1; protarsomere 5 slightly longer than 3. Mesofemur slightly longer than mesotibia, similar to prothoracic legs; mesotarsomere 1 slightly longer than 3, 3 slightly longer than 2, 5 slightly longer than 1. Metafemur gradually enlarging towards second third. Metatibia straight, expanded at apical region; metatibial apex with anterior margin obliquely truncate, posteriorly ascending by one third of apical width; setal comb with setae similar in length along apex, longer along ascending region; articular surface (Fig. 3D, as; surrounding tarsal condyle) densely and completely covered with scales; both inner flange (Fig. 3D, if) and outer bevel (Fig. 3D, ob) fringed by row of spines (“corbel enclosed”). Tarsal claws paired, separate, simple.

**Elytra.** (Fig. 3A, B) 1.6–1.8-times longer than greatest width; greatest width near mid-length in males, near posterior third in females; anterior margins jointly similar in width to posterior margin of pronotum, recessed along median third (projected along striae 1–4, corresponding to emargination of posterior margin of pronotum); humeri oblique, slightly produced; joint elytral width at humeri nearly 1.5-times wider than joint anterior elytral margin, slightly narrower than maximum width of elytra; lateral margins straight and slightly diverging along anterior half to two-thirds, then evenly curved and gradually converging along posterior third; posterior margins each rounded, forming right angle (Fig. 3D). Elytra in lateral view (Fig. 3B) with dorsal outline subplane to slightly convex along anterior half; posterior declivity gradual, evenly convex. Elytra with longitudinally-aligned punctures forming nine complete striae and one incomplete stria (striae 9 and 10 (Fig. 3B, s9, s10) fused near metacoxal insertion); striae narrower than interstriae; punctures separated by distance nearly twice longer than width of each puncture; each puncture with one scale or mostly covered by scales; interstriae 3, 5, 7 (Fig. 3D, i3, i5, i7) and 9 (Fig. 3B, i9) convex along most or part of their length; interstriae 1, 2, 4, 6 and 8 flat to only slightly convex, 10 strongly convex at level of metaventrite; setae similar in length to width of interstriae, recurvate, evenly distributed along interstriae, slightly denser along apical areas; scales covering entire integument, oval, overlapping, uniformly coloured.

**Hind wings.** (Fig. 5D) Fully developed, elongate, nearly 1.4-times longer than elytra, nearly 3.4-times longer than wide; costal margin slightly sinuate along basal half, evenly and broadly curved thereafter; apex narrowly rounded, posterior margin broadly rounded along proximal third; anal lobe not demarcated by emargination of posterior margin of wing. Alar venation well developed: subcosta (Sc), radial (R) and radial recurrent (Rr) well defined, proximal radial sclerite (prs) well developed, darkened, distal radial sclerite (drs) reduced; radial window (w) strongly reduced; radial 3 (R3) very weakly defined (as translucent line), almost reaching alar margin; post radial stripe (pst) darkened, well-defined, almost reaching alar margin; posterior part of postradial stripe (ppp) extending along apical region of wing, nearly one third length of pst, almost reaching alar margin; apical hook of medial stripe (h) conspicuous, nearly one third length of ppp, almost reaching alar margin; medial recurrent (Mr), cubital (Cu) and branch 1 of Cu (Cu1) well defined; crossvein between radial system (rm) very weakly defined; radial sclerotisation (rsc) and medial sclerotisation (msc) weakly defined; medial stripe (mst) weakly defined, slightly shorter than pst; apical fold (af) distinct, nearly reaching alar margin; branch 2 of Cu (Cu2) reduced, smaller than h, not reaching alar margin; anal vein 2 (2A) well defined, vanishing near margin; anal vein 3 (3A) short, reaching mid-length towards margin of wing, fusing with 2A to form anal cell (ac); anal vein 4 (4A) lightly sclerotised, surounded by lightly sclerotised area around base; anal area with margin broadly rounded; posterior margin of hind wing fringed by short and fine setae separated from each other by distance similar to length of one seta, fringe extending to apical fold .

**Abdomen.** Abdominal ventrites (Fig. 3C, E) densely covered with scales; ventrite 1 nearly as long as thoracic metaventrite, with posterior margin sinuate; abdominal ventrite 2 0.7-times as long as 1, nearly as long as 5; surface of ventrites 1 and 2 nearly flat in males (Fig. 3E), convex in females (Fig. 3C); ventrite 5 1.7-times wider than long, with broadly rounded apex in males (Fig. 3E), 1.5-times wider than long, with rounded apex in females (Fig. 3C).

**Male terminalia.** Tergite 7 1.3-times wider than long, with apical corners broadly rounded and broadly emarginate at apex; posteromedial and marginal regions darkened, with relatively thick setae; basi-medial and basi-lateral areas with fine, appressed spines. Tergite 8 subquadrate, only slightly wider than long, with apical corners broadly rounded and truncate to slightly emarginate at apex, with relatively long and thick setae throughout, except along basal fourth; apical margin in posterior view broadly triangularly emarginate. Sternite 8 (Fig. 6A) composed of two lateral somewhat rhomboid sclerites joined medially by membrane, slightly more sclerotised along distal half, with membranous spiculum relictum (Fig. 6A, spr); surface of each sclerite with somewhat coarse punctation; lacking setae. Sternum 9 (spiculum gastrale; Fig. 6B) including apodeme, 1.5-times longer than median lobe, posteriorly bifurcate, furcal arms (Fig. 6B1) opposed, somewhat lanceolate, margins slightly irregular along distal region, posteriorly diverging; each furcal arm basally broad, oval and coarsely punctate, with somewhat triangular distal region, more strongly sclerotised, roundly angulate at apex. Tegmen (Fig. 6C; gonocoxites I, see Boudinot 2018) with apodeme slightly longer than median lobe; tegminal plate with two elongate projections (Fig. 6C, C1), each nearly 0.1-times length of apodeme, finely and densely denticulate along distal half. Aedeagus with median lobe (Fig. 6D; penial sclerite, see Boudinot 2018) 4-times longer than wide; basi-ventral margin broadly emarginate; lateral margins subparallel, apex broadly oval (Fig. 6F), distance from apical margin of ostium (gonopore) to apex of median lobe slightly longer than greatest width of apical region of median lobe; medial lobe in lateral view (Fig. 6E) broadly curved by about 130° basad of mid length, approximately 7.7-times longer than greatest width; greatest width uniform along second third, gradually narrowed along distal third. Endophallus (internal sac) with dense, fine, small papillae (Fig. 6D); with elongate, dorsal, mesal endophallite (Fig. 6F, me) at ostium and ventral membranes (Fig. 6F, vm) projecting lateral and distally forming lateral, elongate, oblique and converging sclerotised regions; basal endophallite complex (see Génier 2019) formed by two basilateral (Fig. 6G, ble) and a mesal (Fig. 6G, bme) rod-like endophallites, followed distally by irregular, transversally folded lamina (Fig. 6G, la) and two lateral bars (Fig. 6G, lb), apically expanding, converging and gradually weakly sclerotised. Aedeagal apodemes (gonocoxites II, see Boudinot 2018) nearly as long as median lobe, slender, slightly broader along anterior half, either fused or very closely articulated to median lobe.

**Female terminalia.** Tergite 7 1.5-times longer than wide, roughly obovate, with anterior and posterior margins broadly and uniformly round, posterior margin more narrowly so; surface sparsely covered by setae, relatively thicker along apical margin; basi-lateral areas with fine, appressed spines. Tergite 8 greatest length 1.2-times longer than greatest width, with anterior margin mesally deeply emarginate; surface gradually more coarsely punctate towards apex, each puncture bearing one seta, setae shorter along apical margin. Sternite 8 (Fig. 7A, B) with apodeme nearly 2.7-times longer than lamina; lamina sagittate, medially divided at apex, 1.7-times longer than wide, apical third covered by setae, gradually more dense towards apex; median region of lamina with mesal paired slightly more sclerotised longitudinal regions; lateral areas of lamina bent upwards (Fig. 7B). Coxites cylindrical (not laterally compressed), nearly 1.6-times longer than lamina of sternum 8; dorsal surface of coxites transversally indented at apical third (Fig. 7; indentation (ind) delimits distal coxites (dcx) from proximal coxites (pcx)); ventral surface of coxites medially longitudinally membranous, areas adjacent to medial membrane darkened; apical margin of coxites in lateral view sinuate, with dorsal section (Fig. 7C, ds) slightly larger and more round in outline than ventral section (Fig. 7C, vs); ventral section of distal coxites with scarce and thick setae; styli (Fig. 7C, sty; 6F) short, tubular, 3-times longer than wide, apically with eight stout setae of two sizes, longer setae pre-apically situated, shorter setae at apex; styli inserted on dorsal lobe of coxites. Genital chamber slightly longer than sternum 8 including apodeme, with a pair of oval, laminar and concave sclerotisations (Fig. 7C, scl) basad of coxites. Membranes of bursa copulatrix (Fig. 7D, bc) papillate. Spermatheca (Fig. 7D, spe; 6E) hook-shaped; ramus (Fig. 7E, ra) longer than collum (Fig. 7E, cl), both apically truncate; corpus (Fig. 7E, crp) shorter and nearly 1.4-times wider than cornu (Fig. 7E, cn); cornu broadly arcuate, apically roundly acute.

##### Variation

Beyond variation in colouration (two colour morphs: green with green with pink/coppery head and legs or completely white to pale brown; see Figs 1, 3) and size (8–15 mm), the morphology of *C.auricephalus* is fairly homogeneous across the distributional range of the species in the U.S. The morphology of both the male (Fig. 6) and female (Fig. 7) genitalia is also highly conserved within the species with no noticeable differences between colour morphs, which confirms their conspecificity.

Champion (1911) highlighted that specimens from southern populations (e.g. Southern Mexico (Acapulco), Guatemala, Costa Rica, Nicaragua and Panama) were uniformly white, had a more elongate body, more produced and acuminate elytral apices and strongly costate alternating interstriae. We studied specimens with these features (Fig. 8A–B) from Costa Rica, Guatemala, Honduras, Nicaragua and Panama, including specimens studied by Champion himself; we also dissected three specimens from Panama (Fig. 8C–E). In general, the specimens, considered by Champion as a variety of *C.auricephalus*, are externally similar to the northern white form and have overall similar male genitalia, but differ clearly by the following characters: brighter white colouration (Fig. 8A, B; as opposed to pearly white to pale brown), cylindrical pronotum (Fig. 8A; as opposed to trapezoid in *C.auricephalus*, see Fig. 3A), conspicuously strongly costate interstriae (in comparison with moderately elevated in *C.auricephalus*), angulate elytral declivity (Fig. 8B; as opposed to broadly rounded in *C.auricephalus*, see Fig. 3B) and acuminate elytral apices (Fig. 8B; as opposed to rounded in *C.auricephalus*, see Fig. 3B). Furthermore, there are evident differences in the shape of the median lobe: median lobe pre-apically widened (Fig. 8C; as opposed to parallel-sided throughout as in *C.auricephalus*, Fig. 6D); in lateral view, median lobe weakly and rather evenly curved (Fig. 8D; as opposed to broadly and strongly curved basad of mid length, in *C.auricephalus*, Fig. 6E), greatest width uniform along median three-fifths (Fig. 8D; as opposed to greatest width uniform along second third, in *C.auricephalus*, Fig. 6E); in dorsal view, gonopore pear-shaped (Fig. 8C; as opposed to oval, as in *C.auricephalus*, Fig. 6D); basal endophallite complex with different configuration (compare Fig. 8E to Fig. 6G).

Based on this character combination (external and male genitalia), we conclude that *Compsusauricephalus* proper extends as far south as Zacapa, Guatemala (CMNEN00019741) and specimens of the Champion (1911) series housed at the BMNH from Costa Rica (Boca del Limón - BMNH(E)1722205; Cangrejal del Aserri - BMNH(E)1722203), Guatemala (Vera Paz - BMNH(E)1722199); Nicaragua (Chontates - BMNH(E)1722202), Panama (Peñas Blancas - BMNH(E)1722207; BMNH(E)1722197; Caldera - BMNH(E)1722204) do not correspond to *Compsusauricephalus*, but to a different species of *Compsus* that may or may not be described with a different name. The Acapulco, Mexico specimen, identified by Champion as part of this variety of *C.auricephalus* (BMNH(E)1722200), is here confirmed to belong to *C.auricephalus* proper. Specimen codes and associated data can be found in Suppl. material 1.

Until more studies can be performed on a broader representation, both across the geographic range and number of specimens of Champion's variety, along with a revision of a larger sample of *Compsus* species, we refer to this variety as *Compsusaff.auricephalus* as defined by Champion (1911) and refrain from naming it as new, given that, even though we have made comparisons with some similarly-looking species, we have not seen all the white species of *Compsus*, nor have we been able to dissect the ones we have examined externally.

#### Distribution

The distributional information for *C.auricephalus* was summarised by O'Brien and Wibmer (1982). The distributional data gathered here are mapped in Fig. 9. The updated distribution is as follows:

***Compsusauricephalus***: Guatemala, Mexico, U.S.A.: Alabama (new record), Arizona (new record), Arkansas, Colorado, Florida (new record; one specimen with incomplete data: just "FLA." on label, USNM, needs confirmation), Georgia (one specimen with incomplete data: just "GA" on label, USNM, needs confirmation), Illinois (Blatchley 1925 [overlooked by O'Brien and Wibmer 1982]), Kentucky (new record), Louisiana, Mississippi, New Mexico (new record), Ohio (new record; incomplete data: just "Ohio" on label, USNM, needs confirmation), Oklahoma (Fenton 1944 [overlooked by O'Brien and Wibmer 1982]), Tennessee (new record), Texas, Utah (new record). Canada: Ontario (intercepted at port of entry; McNamara 1991).

***Compsusaff.auricephalus***: Costa Rica, Honduras (new record), Nicaragua, Panama.

**Comments on current distribution of *Compsusauricephalus*.** According to our specimen examination, *C.auricephalus* occurs north of Zacapa, Guatemala, as far west in the U.S. as Cedar City, Utah and Phoenix, Arizona, as far north as Ohio and as far east to Georgia and Florida. Most of the records are concentrated around Brownsville, Texas and along the Mississippi River. Across the U.S.A., the distribution of *C.auricephalus* seems to be associated with commodities such as cotton plantations (see https://en.wikipedia.org/wiki/Cotton_production_in_the_United_States). There seems to be no geographic pattern of green/white colour morphs.

Records for Florida, Georgia and Ohio (eastern outliers) are represented by single specimens with minimal information: "FLA.", "Ga." and "Ohio", respectively. We report these records here, but highlight that they need confirmation, as these might be cases of interceptions at ports of entry (e.g. Florida), incomplete information (e.g. there is an "Ohio" locality in Hamilton Co., Texas, which is in the area where the species has been recorded) or just mislabelling.

Western outliers (Arizona, Colorado, New Mexico and Utah) are represented by either records from literature (see O'Brien and Wibmer 1982) or single specimens in collections. Given that there are unique records for each of these occurrences and, even though they have more detailed collecting information (see GBIF dataset at https://doi.org/10.15468/dl.rat633 and Suppl. material 1), they may be adventitious (e.g. transported with plants from elsewhere) and not part of actual established populations of the species in those regions. Data from local collections, as well as more sampling from those areas, would be required to confirm the presence of *C.auricephalus* in those States.

As for *Compsusaff.auricephalus*, it is known to occur from Yoro, Honduras, south to the Canal Zone in Panama (Fig. 9).

#### Ecology

*Compsusauricephalus* has been collected in palmetto thickets and woods (Blatchley and Leng 1916) and, according to label data, on vegetation along roads, on prairies and using a variety of methods (beating, blacklights, flight intercept traps, malaise traps, pitfall traps, sweeping, manual capture). The species has been found at elevations from sea level up to 1200 m, with most specimens collected below 100 m.

##### Associated plants

Host plant records were recovered from literature, as well as from label data from specimens in collections. Host specificity in broad-nosed weevils is difficult to assess, given that the presence of adults on a particular plant does not necessarily mean that feeding occurred (Kissinger 1964). A summary including 46 plant species in 23 families is presented in Table 1.

#### Biology

According to Blatchley and Leng (1916), citing Pierce (1916), *Compsusauricephalus* "lays its eggs in a mass of gummy substance on leaves and folds a portion on the leaf over them so that they are perfectly concealed. [...] The eggs hatch in seven or eight days and the larvae enter the ground to feed on the roots of plants." This behaviour matches Howden's oviposition category 9: eggs and adhesive placed in fabricated or discovered niches, usually without use of rostrum, which is typical of most "Adelognatha" (Howden 1995). "The larvae are typical legless, C-shaped, white to translucent grubs that feed on roots in soil [...] adults are collected in June and July in southern Texas (Headrick 2014).

According to Headrick (2014), damage caused by *C.auricephalus* on *Citrus* is significant in Texas; most of the damage is associated with the underground larval stages which open the door for *Phytophthora* (Oomycota) infections to the trees; the habits of the larvae make control measures difficult and costly. In addition, *C.auricephalus* has been reported to damage young cotton plants in Arkansas, Oklahoma, Tennessee and Texas (Agricultural Research Service, United States Department of Agriculture 1978).

As for natural enemies, *Tetrastichuscompsivorus* Crawford, 1914 (Hymenoptera: Chacidoidea, Crawford 1914) has been recorded from eggs of *C.auricephalus* in Oklahoma (Crawford 1914), as well as from Texas, Mississipi (Krombein et al. 1979) and Nicaragua (Maes and O'Brien 1990). There are mentions of Gray Grosbeak (*Pyrrhuloxiasinuata*) and Cardinal (*Cardinaliscardinalis*) feeding on miscellaneous beetles including *C.auricephalus* (McAtee 1908).

##### Barcode data

Molecular data from the mitochondrial gene Cytochrome Oxidase subunit I (COI) was obtained for the specimen identified as USNMENT01070595 (see Suppl. material 1). The sequence of 361 bp is identified with the GenBank number MN344151.1 (https://www.ncbi.nlm.nih.gov/nuccore/1770599749).

#### Taxon discussion

Due to its variation in size and colouration (two main colour morphs: predominantly green with pink/coppery head and part of the legs or completely white to pale brown), *Compsusauricephalus* can be confused with a number of eustyline species from Central and South America, not only with other *Compsus* species, but with species of other genera in the *Compsus* genus complex in South America and even species in the *Exophthalmus* genus complex in Central America. Colouration patterns alone partly overlap with at least a dozen different species; in these instances, particularities of the colouration pattern (e.g. colouration of legs and/or elytra) along with characters of the elytral sculpture allow for differentiation. Here, we illustrate and discuss some characters useful for distinguishing *C.auricephalus* from look-alike species.

*Compsusadonis* Marshall, 1922 (Marshall 1922) is a brightly-coloured species with the head, anterior margin of the pronotum, apical region of elytra and outer surfaces of the tibia covered with pink scales, with the elytra, remainder of prothorax and elytra and femora mostly green; the apices of the femora and the tarsi are covered by blue scales and the elytra have irregular areas covered by white scales blending in with the general green coverage (Fig. 10B). This very particular colouration pattern is quite different from the colourations exhibited by *C.auricephalus*. In addition, the less regularly distributed elytral punctation, along with the irregularly-elevated surface of the elytra and projected elytral apices in *C.adonis* further allows its recognition. *Compsusadonis* is endemic to Colombia (Wibmer and O'Brien 1986).

**Figure 2.**
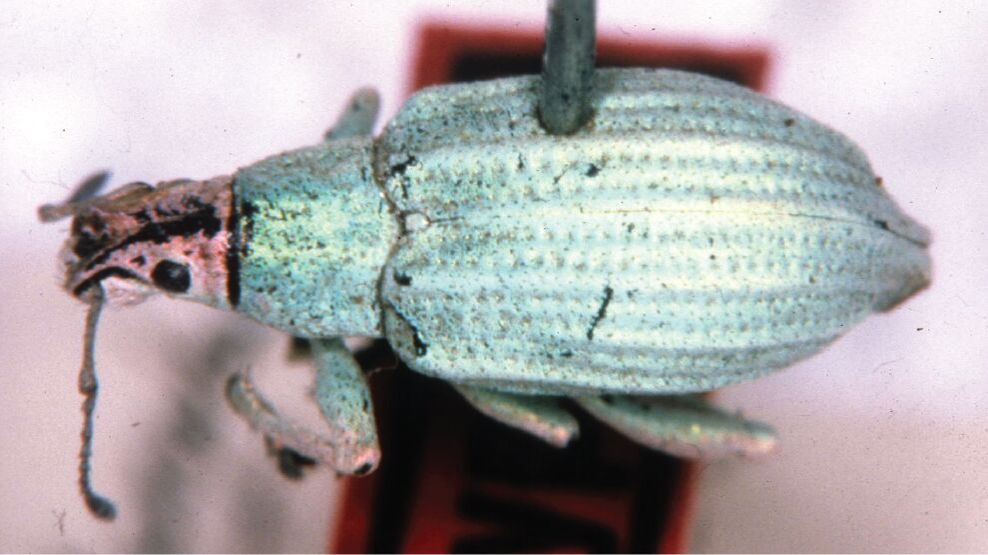
Holotype of *Platyomusauriceps* Schönherr, 1840. Courtesy of M. Guadalupe del Río, taken at NHRS.

**Figure 3.**
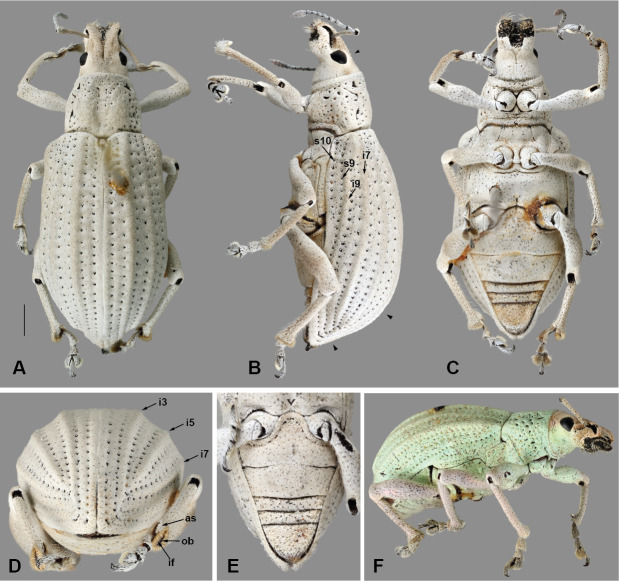
Morphological features of *Compsusauricephalus*: (A–D) Texas, Garza Co. (TTU-Z_050014); scale bar: 1 mm: (A) dorsal habitus, (B) lateral habitus (s10: stria 10; s9: stria 9; i7: interstria 7; i9: interstria 9), (C) ventral habitus of female, (D) posterior view (i3: interstria 3; i5: interstria 5; i7: interstria 7; as: articular surface; ob: outer bevel; if: inner fringe), (E) Texas, Cameron Co. (TTU-Z_219291) ventral habitus of male; (F) Mississippi, Warren Co. (TTU-Z_219296), anterolateral habitus.

**Figure 4.**
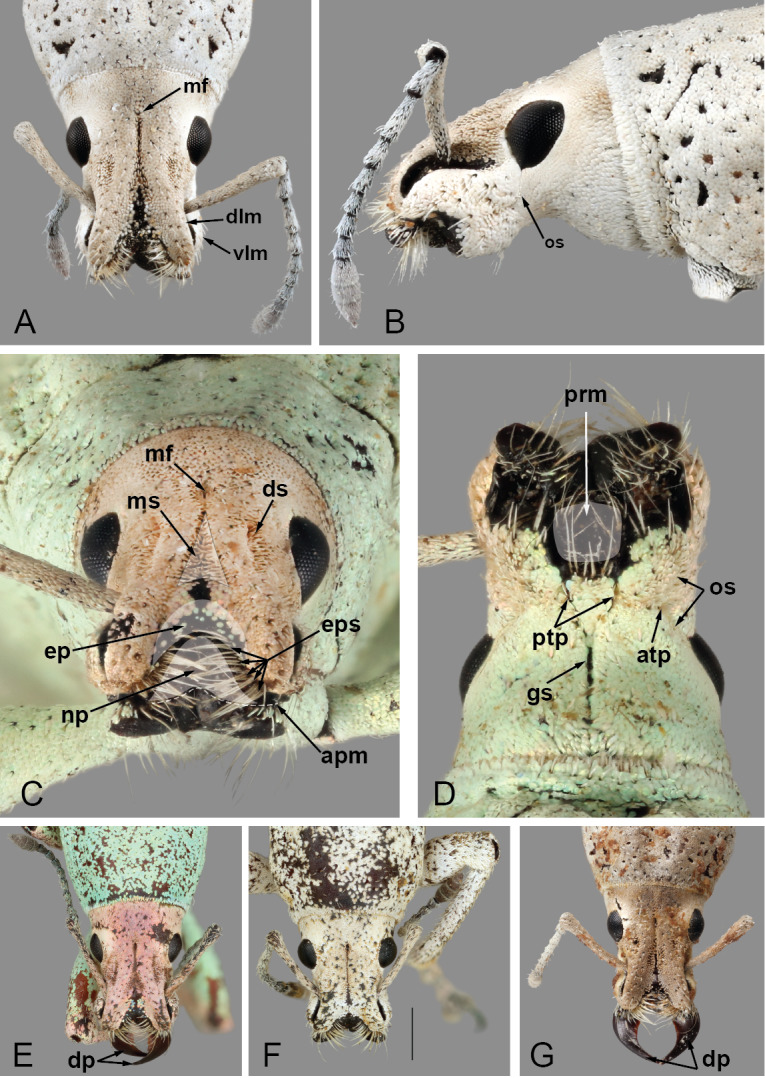
Morphological features of the head of *Compsusauricephalus*: (A–B) Texas, Garza Co. (TTU-Z_050014): (A) dorsal view (mf: median fovea; dlm: dorsolateral margin; vlm: ventrolateral margin), (B) lateral view (os: occipital suture); (C–D) Mississippi, Warren Co. (TTU-Z_219296): (C) head, anterior view (mf: median fovea; ms: median sulcus; ds: dorsolateral sulcus; ep: epistoma; eps: epistomal setae; np: nasal plate; apm: apical margin), (D) head, ventral view (prm: prementum; os: occipital suture; atp: anterior tentorial pit; ptp: posterior tentorial pits; gs: gular suture), (E) Mississippi, Washington County (CWOC0045), dorsal view with deciduous processes (dp); (F) Texas, Brooks County (CWOC0002) dorsal, scale bar 1 mm; (G) Texas, Mason Co. (TTU-Z_219308), dorsal view with deciduous processes (dp).

**Figure 5.**
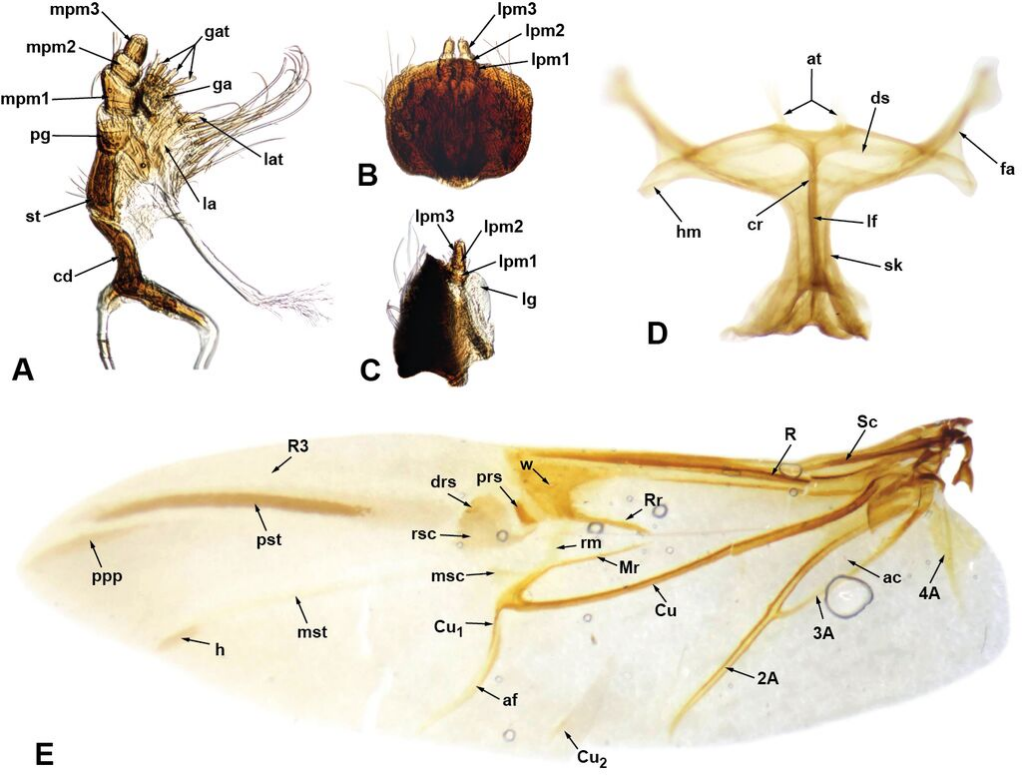
Morphological features of *Compsusauricephalus* (Louisiana, Acadia Parish): (A–C) mouthparts: (A) right maxilla, ventral view (cd: cardo; st: stipes; pg: palpiger; mpm1: maxillary palpomere 1; mpm2: maxillary palpomere 2; mpm3: maxillary palpomere 3; gat: galeal teeth; ga: galea; lat: lacinial tooth; la: lacinia), (B–C) prementum, (B) dorsal view, (C) lateral view (lpm1: labial palpomere 1; lpm2: labial palpomere 2; lpm3: labial palpomere 3; lg: ligula), (D) metendosternite, posterior view (sk: stalk; fa: furcal arm; cr: crux; ds: distal sheath; at: anterior tendons; lf: longitudinal flange; hm: hemiductus), (E) hindwing (Sc: subcosta; R: radial; Rr: radial recurrent; rm: crossvein between radial system and Mr; Mr: medial recurrent; w: radial window; prs: proximal radial sclerite; rsd: distal radial sclerite; rsc: radial sclerotisation; msc: medial sclerotisation; Cu: cubital; Cu1: branch of Cu; af: apical fold; Cu2: branch of Cu; 2A: anal vein 2; 3A: anal vein 3; ac: anal cell; 4A: anal vein 4; R3: radial 3; pst: postradial stripe; ppp: posterior part of postradial stripe; h: apical hook of medial stripe; mst: medial stripe).

**Figure 6.**
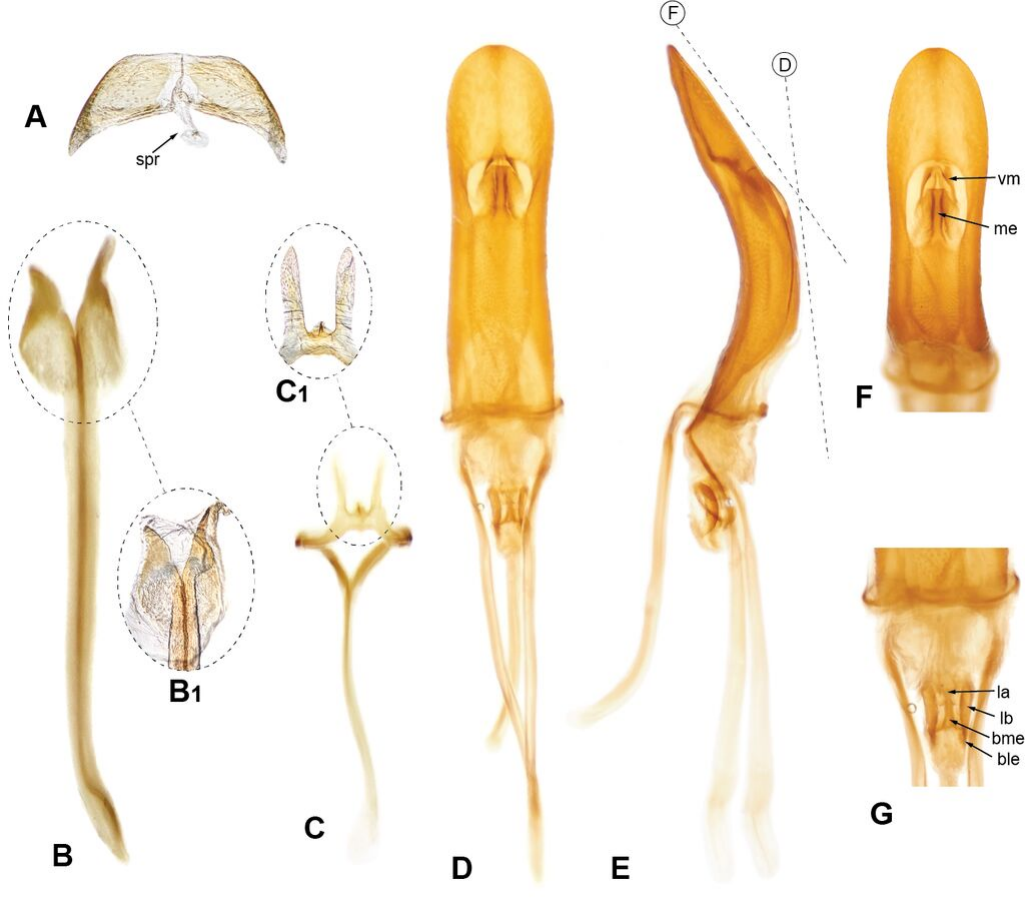
Male genitalia of *C.auricephalus*: (A) abdominal sternite 8 (spr: spiculum relictum), (B) spiculum gastrale, (B1) detail of furcal arms, (C) tegmen, (C1) detail of projections of tegminal plate, (D) aedeagus, dorsal view (see dashed line D in figure 5E), (E) aedeagus, lateral view with dashed lines D and F indicating plane for figures D and F, respectively, (F) apical region of aedeagus, dorsal view (see dashed line F in figure 5E; me: mesal endophallite, vm: ventral membranes), (G) detail of basal endophallite complex, dorsal view (la: distal irregular lamina, lb: distal lateral bars), bme: basimesal endophallite, ble: basilateral endophallites).

**Figure 7.**
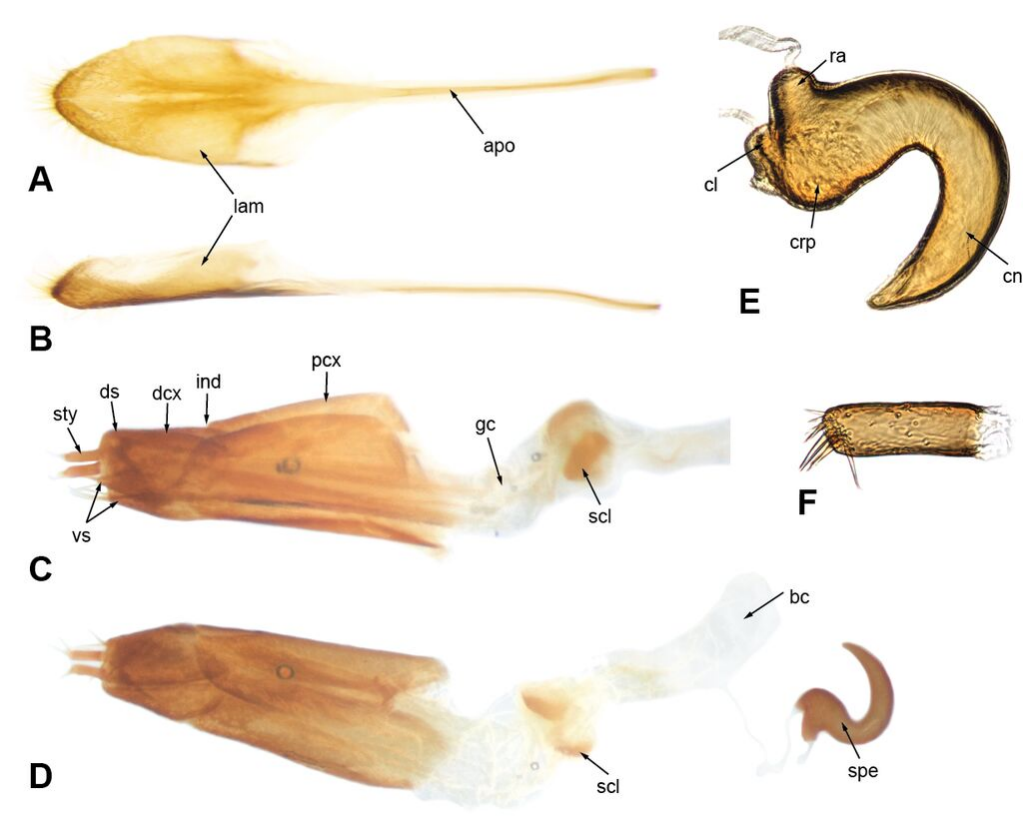
Female genitalia of *C.auricephalus*: (A–B) abdominal sternite 8 (apo: apodeme; lam: lamina), (A) ventral view, (B) lateral view, (C–D) ovipositor, (C) coxites, lateral view (sty: stylus; vs: ventral section of distal coxites; ds: dorsal section of distal coxites; dcx: distal coxites; ind: indentation; pcx: proximal coxites; gc: genital chamber; scl: sclerites) (D) dorsal view (scl: sclerites; bc: bursa copulatrix; spe: spermatheca), (E) spermatheca (ra: ramus; cl: collum; crp: corpus; cn: cornu), (F) detail of stylus.

**Figure 8.**
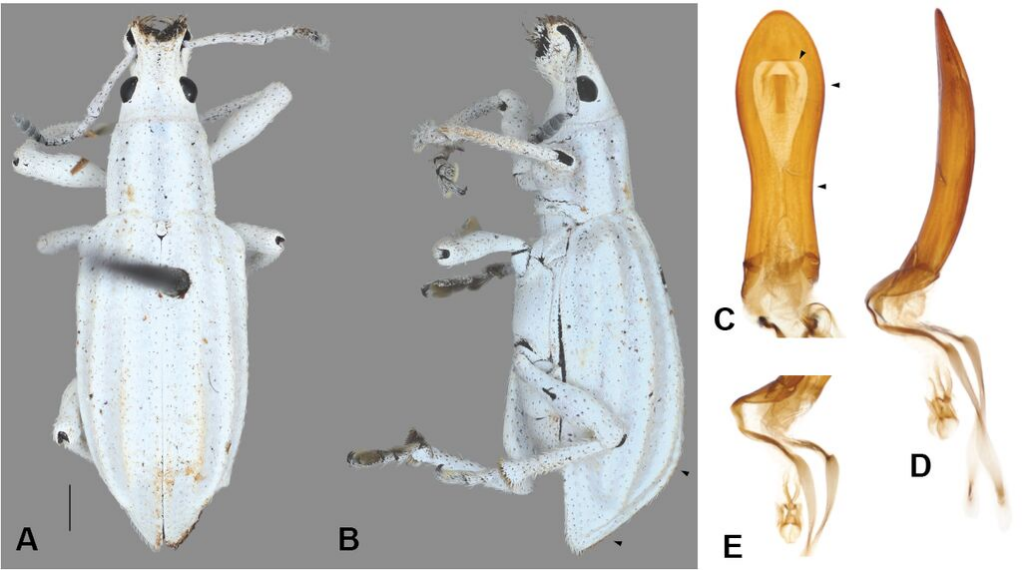
Compsusaff.auricephalus, Panama, Cerro Campana: (A–B) habitus, scale bar 1 mm: (A) dorsal, (B) lateral, (C) median lobe, dorsal view, (D) aedeagus, lateral view, (E) detail of basal endophallite.

**Figure 9.**
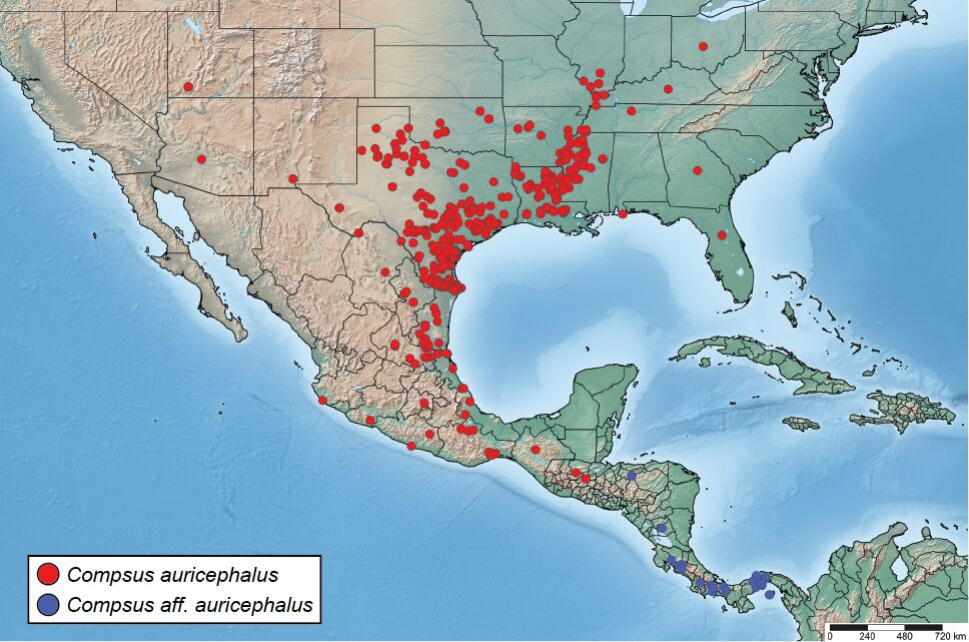
Map of localities recorded for *Compsusauricephalus* and *Compsusaff.auricephalus*.

**Figure 10.**
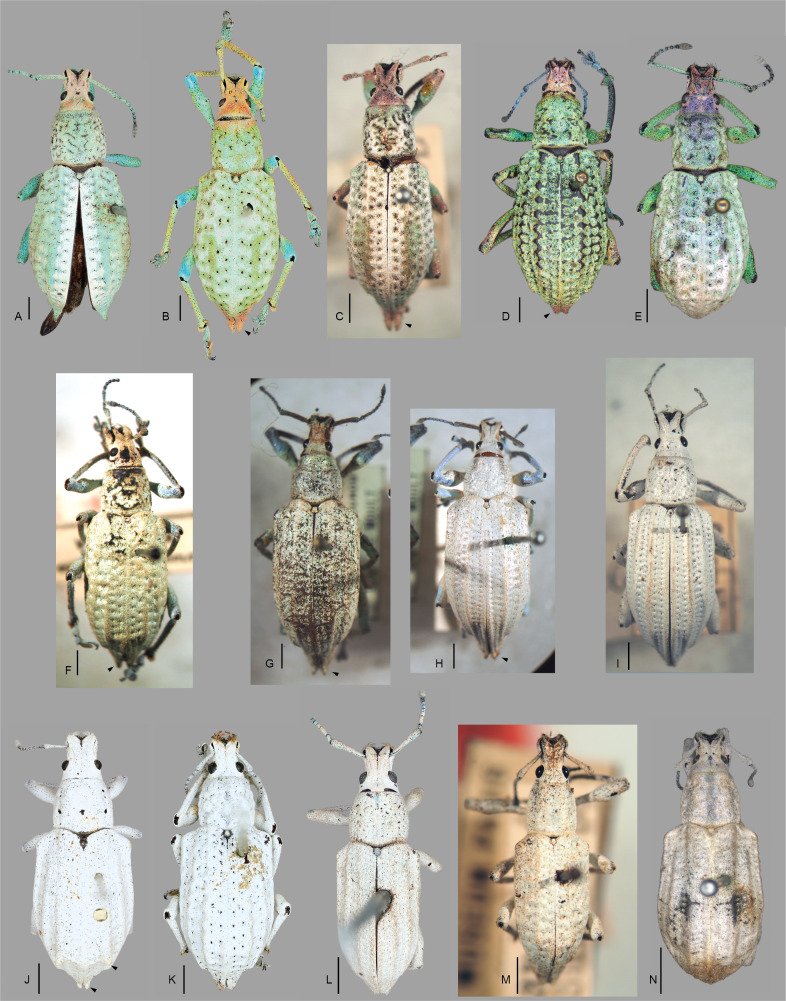
Habitus of species in the *Compsus* genus complex apparently similar to *C.auricephalus*: (A) *Oxydercesviridipes* NHRS-JLKB000022891 (syntype, NHRS), (B) *Compsusadonis* BMNH(E)1722340 (holotype, NHMUK, (C) *Compsusbellus* (holotype, MNHN), (D) *Oxydercesexaratus* (syntype, MNHN), (E) *Compsusviolaceus* (syntype, MNHN), (F) *Compsussulcicollis* (holotype, MNHN), (G) *Compsuslacteus* (MNHN, white form), (H) *Compsuslacteus* (MNHN, green form), (I) *Compsusalbus* (syntype MNHN), (J) *Oxydercescretaceus* ZMUC00037512 (holotype, ZMUK), (K) *Oxydercesmansuetus* NHRS-JLKB000023017 (paratype, NHRS), (L) *Compsusexanguis* NHRS-JLKB000022886 (syntype, NHRS), (M) *Compsusvirginalis* (syntype, MNHN) and (N) *Oxydercescinereus* (syntype, MNHN). Scale bars: 1 mm.

*Compsusalbus* Hustache, 1938 (Hustache 1938) shares many of the characteristics present in specimens of the white form of *C.auricephalus*, as well as with *C.aff.auricephalus*, as defined by Champion (1911): body covered by uniformly-white scales, costate interstriae 3, 5 and 7, acuminate elytral apices with regularly-aligned elytral punctures. The holotype of this species has a shiny lustre throughout (Fig. 10I). *Compsusalbus* can be differentiated by the lack of a medial sulcus along the rostrum, which is present in *C.auricephalus* (compare Fig. 10I vs. Figs 2, 3A, C). *Compsuscandidus* is similar to *C.albus*, both species being endemic to Colombia (Wibmer and O'Brien 1986).

In *Compsusbellus* Hustache, 1938 (Hustache 1938), the head and distal parts of the legs are covered by coppery/pink scales just as in the green form of *C.auricephalus*. *Compsusbellus* can be recognised by its combined green and white thorax and elytra, where the elytra have a broad white medial longitudinal stripe (Fig. 10C; as opposed to either uniformly-green or uniformly-white thorax and elytra in *C.auricephalus*, see Figs 1, 3) and by the strongly-projected elytral apices (Fig. 10C; apex of elytra not projected in *C.auricephalus*, see Fig. 3A). In addition, there are no conspicuously-elevated interstria along the elytra in *C.bellus*. Even though *C.bellus* is currently considered a junior synonym of *C.adonis*, by comparing both holotypes, beyond the evident differences in colouration (compare Fig. 108B vs. Fig. 10C), the elytral punctation and the length of the apical elytral projections suggest that these are, in fact, different species, although, until more detailed studies, including dissections of male and female genitalia can be performed, we refrain from making any taxonomic changes at this time. *Compsusbellus* is endemic to Colombia (Wibmer and O'Brien 1986).

*Compsusdivisus* Hustache, 1938 (Hustache 1938) has the head and entire legs (except the tarsi) covered by pink scales, with bluish-green scales covering thorax and elytra; it also has part of the odd-numbered elytral interstriae elevated. The round eyes in lateral view of the head, along with the presence of an elevated, glabrous and smooth longitudinal ridge along the head and rostrum would allow the recognition of *C.divisus*; this longitudinal ridge suggests that this species may actually be better placed in the genus *Exophthalmus* (Franz 2012, Zhang and Franz 2015). *Compsusdivisus* is endemic to Colombia (Wibmer and O'Brien 1986).

*Compsusexanguis* (Boheman, 1833) (Schönherr 1833) is generally similar to the white form of *C.auricephalus*, except for the conspicuous thick setae uniformly distributed all over the surface of the body and the presence of green scales at midpoint of the anterior margin of the pronotum, scattered through the antennae, at the apex of the tibiae and along the tarsi (Fig. 10L). *Compsusdivisus* is endemic to Peru (Wibmer and O'Brien 1986).

*Compsuslacteus* (Fabricius, 1781) (Fabricius 1781) has variable colour morphs within the same colour range as *C.auricephalus* (i.e. green and white colour forms); however, the head is never pink and the legs and antennae are always distally blue (Fig. 10G, H). *Compsuslacteus* has strongly-projected eyes, projected elytral apices and conspicuous setae along the surface of the prothorax and elytra, which differentiates it from *C.auricephalus*. *Compsuslacteus* is distributed in Brazil, French Guiana, Guadeloupe and Jamaica (O'Brien and Wibmer 1982, Wibmer and O'Brien 1986).

*Compsussulcicollis* Hustache, 1938 (Hustache 1938) has the head covered by coppery/pink scales, with the legs covered by light green and blue scales; the thorax and elytra are uniformly covered by light green scales; the surface of elytral interstriae 3 is elevated only along the elytral declivity in *C.sulcicollis* and the apices of the elytra are strongly projected (Fig. 10F); these features allow its differentiation from *C.auricephalus*. *Compsussulcicollis* is endemic to Venezuela (Wibmer and O'Brien 1986).

*Compsusviolaceus* Hustache, 1938 (Hustache 1938) follows the general pink head/green body pattern of the green form of *C.auricephalus*; however, the rostrum of *C.violaceus* is covered by pink scales, while the frons and mid-anterior section of the pronotum are covered by purple scales; the remainder of the pronotum and anterior half of the elytra are covered by pale green scales, the posterior half of the elytra are covered mostly by white scales and the legs are covered completely by iridescent green scales (Fig. 10E). In addition, the overall body shape of *C.violaceus* is pyriform (Fig. 10E), as opposed to subrectangular as in *C.auricephalus* (Fig. 3A). *Compsusviolaceus* is endemic to Colombia (Wibmer and O'Brien 1986).

*Compsusvirginalis* Faust, 1892 (Faust 1892) is generally similar to the white form of *C.auricephalus* and more so to *C.aff.auricephalus*, as defined by Champion (1911), as it is uniformly covered by white scales, has cylindrical prothorax and acuminate elytral apices. It can be distinguished by its somewhat irregular elytral punctation and irregularly-elevated elytral surface (Fig. 10M; as opposed to having costate odd-numbered elytral interstriae). *Compsusvirginalis* is endemic to Venezuela (Wibmer and O'Brien 1986).

*Exophthalmuscarneipes* Champion, 1911 (Champion 1911), *E.cupreipes* Champion, 1911 (Champion 1911), *E.opulentus* Boheman, 1840 (Schönherr 1840) and *E.vitticollis* Champion, 1911 (Champion 1911) are generally similar to the green form of *C.auricephalus*, all of them being predominantly covered by green scales with the head covered by pink scales. The legs fully covered by pink scales and the presence of an elevated, glabrous and smooth longitudinal ridge along the head and rostrum clearly differentiate *C.auricephalus* from these *Exophthalmus* species. In addition, *E.carneipes*, which is distributed in Costa Rica, Honduras and Panama (O'Brien and Wibmer 1982), has transverse bands of pink scales along the elytral shoulders and elytral apices, (see https://www.inaturalist.org/taxa/874484-Exophthalmus-carneipes). On the other hand, *E.cupreipes*, which is endemic to Mexico (O'Brien and Wibmer 1982), has a more elongated rostrum and its scales are green with golden tones (Bautista-Martínez et al. 2020), very similar to *E.opulentus* from Guatemala and Mexico (O'Brien and Wibmer 1982); these two species can be differentiated by the fully pink head in *E.opulentus* (https://www.inaturalist.org/taxa/269236-Exophthalmus-opulentus), whereas the head of *E.cupreipes* has green lateral areas. *Exopthalmusvitticollis*, from Belize and Guatemala (O'Brien and Wibmer 1982) can be recognised by the presence of a pink median longitudinal stripe along the pronotum (https://www.inaturalist.org/taxa/1073743-Exophthalmus-vitticollis).

*Oxydercescinereus* (Hustache, 1938) (Hustache 1938) is generally similar to the white form of *C.auricephalus*, especially because of its elevated odd-numbered elytral interstriae. It differs by the pyriform shape of the body and the greyish colouration of the scales covering the head and the mid and lateral lines of the pronotum, along with the presence of a pair of relatively-large dark spots about mid-length on the elytra (Fig. 10N). *Oxydercescinereus* is endemic to Ecuador (Wibmer and O'Brien 1986).

*Oxydercescretaceus* (Fabricius, 1792) (Fabricius 1792) is similar to the white form of *Compsusauricephalus* and nearly identical to *C.aff.auricephalus*, as defined by Champion (1911). The sculpture of the pronotum with three basal foveae, the tubercles of interstriae 3 at elytral declivity and the apical projections of the elytra clearly distinguish *O.cretaceus* (Fig. 10J). *Oxydercescretaceus* has been recorded from Guadeloupe and Martinique (O'Brien and Wibmer 1982). Records of "*Compsusauricephalus*" in iNaturalist from Trinidad and Tobago (https://www.inaturalist.org/observations/32103921) may actually correspond to *O.cretaceus*, but the available specimen photographs do not allow its confirmation.

*Oxydercesexaratus* (Hustache, 1938) (Hustache 1938) is similar to the green form of *Compsusauricephalus*, in the presence of pink scales covering the head and green scales covering most of the body. They differ mostly by the presence of blue scales covering the antennae and tarsi and by the rounded projections of the elytral apices which are covered by pink scales (Fig. 10D). In addition, the surface of the elytra is irregularly elevated in *O.exaratus*, in contrast with the elevated odd-numbered elytral interstriae typical of *C.auricephalus* (see Fig. 3A). *Oxydercesexaratus* is endemic to Colombia (Wibmer and O'Brien 1986).

*Oxydercesmansuetus* Hustache, 1938 (Hustache 1938) is similar to the white form of *Compsusauricephalus*, differing by the deep punctation of both pronotum and elytra, resulting in irregularly-elevated elytral surface, especially along the posterior half of elytra in *O.mansuetus* (Fig. 10K). In addition, *O.mansuetus* has strongly projected eyes and acuminate elytral apices (Fig. 10K) and is endemic to Argentina (Wibmer and O'Brien 1986).

*Oxydercesviridipes* Boheman, 1840 (Schönherr 1840) is a species with a very similar colouration pattern to the green form of *Compsusauricephalus*, especially because of the pink colouration of the head. The fully green legs and white longitudinal band along the elytral interstriae 1 and 2, along with the sculpture of the elytra lacking elevated interstriae (Fig. 10A), clearly distinguish *O.viridipes*, which is endemic to Colombia (Wibmer and O'Brien 1986), quite common in Medellín and surrounding areas (see https://www.inaturalist.org/observations?taxon_id=869547). *Oxydercesviridipes* has been found domestically in Pennsylvania and California (https://www.inaturalist.org/observations/6246529) and intercepted at south-eastern ports of entry in the U.S.A.

## Supplementary Material

XML Treatment for Compsus auricephalus

32E48EC3-1CA1-590B-A955-62F1C084D277 10.3897/BDJ.8.e55474.suppl1 Supplementary material 1 Part of material examined from miscellaneous collections Data type Occurrences Brief description This file contains data of specimens from CLEV, CMNC, INHS, LSAM, MEM, NHMUK and USNM, which are currently not available online. It was created using the IPT template available at https://github.com/gbif/ipt/wiki/occurrenceData. Coordinates were approximated via Google Maps based on locality information. File: oo_427863.xlsx https://binary.pensoft.net/file/427863 Girón & Chamorro

## Figures and Tables

**Table 1.** Host plants associated with *Compsusauricephalus*. Acronyms in the References column correspond to collections presented in Materials and Methods. Localities with an asterisk (*) correspond to *Compsusaff.auricephalus*, as defined by Champion (1911).

**Plant family**	**Plant species**	**Locality**	**References**
Amaranthaceae	*Beta vulgaris*[Beet]	Texas	USNM
Asteraceae	*Ambrosia*	Texas	Mitchell and Pierce 1911 Champion 1911, USNM
	*Ambrosiatrifida* [Giant ragweed]	Texas	USNM
	*Baccharisneglecta*[False willow]	Texas	USNM
	*Helianthus*[Sunflower]	Mexico (Nuevo León)	USNM
	*Partheniumhysterophorus* [Santa Maria feverfew]	Mexico (Coahuila, Nuevo León)	Soto-Hernández and Barros-Barrios 2018 USNM
	*Xanthium* [Cocklebur]	Mississippi	MEM
Boraginaceae	*Ehretiaanacua* (formerly *Ehretiaelliptica*) [Knockaway]	Texas	Blatchley and Leng 1916
Cactaceae	*Opuntialeptocaulis*	Texas	USNM
	*Opuntialindheimeri*	Texas	USNM
Cannabaceae	*Celtislaevigata*[Sugarberry]	Texas	USNM
Cornaceae	*Cornussericea* [Red-osier dogwood]	Texas	USNM
Euphorbiaceae	*Ricinuscommunis* [Castor bean]	Texas	USNM
Fabaceae	*Acacia*	Texas	Mitchell and Pierce 1911 Champion 1911, USNM
	*Acaciafarnesiana* [Sweet acacia]	Texas	USNM
	*Baptisianuttalliana* [Nuttall's wild indigo]	Louisiana	MEM
		Texas	VanDyk et al. 2020
	*Lespedeza* [Bush clovers]	Tennessee	USNM
	*Leucaenapulverulenta*[Mexican Leadtree]	Texas	USNM
	*Medicagosativa*[Alfalfa]	Oklahoma	USNM
	*Phaseolusvulgaris* [Bean foliage]	Texas	USNM
	*Prosopis* sp.	Texas	USNM
	*Prosopisjuliflora*	Mexico (San Luis Potosí)	USNM
	*Prosopisglandulosa* [Honey Mesquite]	Texas	Headrick 2014 Museum of Texas Tech University (TTU) 2020
	*Vignaunguiculata* [Black-eyed pea]	Texas	USNM
Fagaceae	*Quercus* [Oak]	Oklahoma	USNM
Gelsemiaceae	*Gelsemiumsempervirens* [Carolina Jessamine]	Mississippi	MEM
Juglandaceae	*Caryaillinoinensis* [Pecan]	Louisiana	USNM
		Mississippi	MEM
		Texas	USNM
		Mexico (Coahuila)	Soto-Hernández and Barros-Barrios 2018
Lamiaceae	*Monardacitriodora* [Lemon beebalm]	Texas	USNM
Lauraceae	*Perseaamericana*[Avocado foliage]	Panama*	USNM
Malvaceae	*Gossypium* [Cotton]	Arkansas	Agricultural Research Service, United States Department of Agriculture 1978
		Louisiana	USNM
		Mississippi	Johnson and Cora 2018, MEM
		Oklahoma	Fenton 1944, Agricultural Research Service, United States Department of Agriculture 1978, USNM
		Tennessee	Agricultural Research Service, United States Department of Agriculture 1978
		Texas	Mitchell and Pierce 1911, Champion 1911, Pierce 1916, Blatchley and Leng 1916, Agricultural Research Service, United States Department of Agriculture 1978, USNM
	*Hibiscus*	Mississippi	MEM
Piperaceae	*Piper* sp.	Texas	USNM
Poaceae	*Sorghumbicolor* [Sorghum]	Mexico (Nuevo León)	USNM
	*Triticum* [Wheat]	Oklahoma	USNM
	*Zea* [Corn]	Mississippi	USNM
		Nicaragua	Maes and O'Brien 1990
Polygonaceae	*Rumexcrispus* [Curly dock]	Texas	USNM
Rosaceae	*Crataegus*[Hawthorn]	Louisiana	USNM
	Fragaria×ananassa [Strawberry]	Tennessee	USNM
	*Malusdomestica* [Apple]	Illinois	Dmitriev 2015
		Mississippi	MEM
	*Prunuspersica* [Peach]	Louisiana	USNM
		Illinois	Dmitriev 2015
		Mississippi	MEM
	*Pyrus* [Pear]	Mississippi	MEM
Rutaceae	*Citrus*	Texas	Woodruff 1985, Headrick 2014, USNM
		Mexico (Nuevo León, Tamaulipas)	USNM
Salicaceae	*Populus* [Cottonwood]	Tennessee	USNM
	*Populusdeltoides*[Eastern cottonwood]	Texas	USNM
Santalaceae	*Phoradendron* [Mistletoe]		Champion 1911
	*Phoradendronflavescens* [Mistletoe]	Texas	Mitchell and Pierce 1911, Tucker 1919 (adventitious), Anderson 1994 (incidental), USNM
Solanaceae	*Nicotianarepanda* [Fiddleleaf tobacco]	Texas	USNM
	*Solanum* [Potatoes]	Mississippi	MEM
		Texas	USNM (on foliage of Irish potatoes)
Verbenaceae	*Lantanacamara* [Wild sage]	Texas	Blatchley and Leng 1916
